# Role of RNA Motifs in RNA Interaction with Membrane Lipid Rafts: Implications for Therapeutic Applications of Exosomal RNAs

**DOI:** 10.3390/ijms22179416

**Published:** 2021-08-30

**Authors:** Rafał Mańka, Pawel Janas, Karolina Sapoń, Teresa Janas, Tadeusz Janas

**Affiliations:** 1 Institute of Biology, University of Opole, Kominka 6, 45-032 Opole, Poland; rafal.manka@uni.opole.pl (R.M.); karolina.sapon@uni.opole.pl (K.S.); teresa.janas@uni.opole.pl (T.J.); 2 Kellogg School of Management, Northwestern University, Evanston, IL 60208, USA; pawel.janas8@gmail.com

**Keywords:** exosomes, FRET spectroscopy, liposomes, RNA aptamers, RNA motifs

## Abstract

RNA motifs may promote interactions with exosomes (EXO-motifs) and lipid rafts (RAFT-motifs) that are enriched in exosomal membranes. These interactions can promote selective RNA loading into exosomes. We quantified the affinity between RNA aptamers containing various EXO- and RAFT-motifs and membrane lipid rafts in a liposome model of exosomes by determining the dissociation constants. Analysis of the secondary structure of RNA molecules provided data about the possible location of EXO- and RAFT-motifs within the RNA structure. The affinity of RNAs containing RAFT-motifs (UUGU, UCCC, CUCC, CCCU) and some EXO-motifs (CCCU, UCCU) to rafted liposomes is higher in comparison to aptamers without these motifs, suggesting direct RNA-exosome interaction. We have confirmed these results through the determination of the dissociation constant values of exosome-RNA aptamer complexes. RNAs containing EXO-motifs GGAG or UGAG have substantially lower affinity to lipid rafts, suggesting indirect RNA-exosome interaction via RNA binding proteins. Bioinformatics analysis revealed RNA aptamers containing both raft- and miRNA-binding motifs and involvement of raft-binding motifs UCCCU and CUCCC. A strategy is proposed for using functional RNA aptamers (fRNAa) containing both RAFT-motif and a therapeutic motif (e.g., miRNA inhibitor) to selectively introduce RNAs into exosomes for fRNAa delivery to target cells for personalized therapy.

## 1. Introduction

Exosomes are small (30–100 nm) membrane nanovesicles originating from endosomes, that are released from the parent cell into the extracellular environment [1]. Their release was reported from various cell types, such as: reticulocytes [1], B and T lymphocytes [2,3], dendritic cells [4] as well as cancer cells [5]. This type of extracellular vesicles (EVs) can be detected in most body fluids including urine, blood plasma, saliva, breast milk, and cerebrospinal fluid [6,7].

Since the first description of microvesicles produced by platelets in human blood and the discovery of their procoagulant properties [8] the role of exosomes is constantly being explored. Their direct function is to transport various molecules such as proteins, lipids, and nucleic acids between cells, and to protect the cargo contained within them. Thus, exosomes mediate cell-to-cell communication, as exemplified by immune cells [9,10] where T cells both receive [2] and send [11] information via exosomes. The involvement of exosomes was also reported in various pathological conditions such as cardiovascular diseases [12], viral infections [13], neurodegenerative diseases [14], and cancer [15,16,17].

Exosomes are formed as intraluminal vesicles (ILVs) by the inward budding of the multivesicular body (MVB) and are secreted into the extracellular space by fusion of MVB with the cell membrane [18]. The membrane of exosomes is enriched with cholesterol, sphingomyelin, phosphatidylserine, and glycosphingolipids (e.g., ceramide) [19,20] as well as GM1 ganglioside [21] in comparison to the cell membrane. Such a lipid composition promotes the formation of lipid rafts, the more organized nanodomains inside membranes.

Membrane rafts are heterogeneous, dynamic, cholesterol and sphingolipid-enriched nanodomains (10–200 nm in size) within membranes, that compartmentalize cellular processes [22] and may form microscopic domains upon clustering [23]. Rafts can regulate the distribution of proteins and adhesion molecules on the surface of exosomes allowing their interaction with membranes of recipient cells [24,25]. Ordered regions of the MVB membrane such as lipid rafts are likely to be involved in the formation of exosomes, independent of endosomal sorting complex required for transport [24,26,27,28,29,30].

Although the exact mechanism of RNA loading into exosomes is still being explored, it appears that exosomal RNAs are not loaded randomly. Studies on RNAs transported by exosomes show that exosomal RNA content may differ from that of the parent cell [31,32,33,34] which suggests that the sorting of specific RNA species to exosomes is rather selective.

The factors involved in the active regulation of the exosomal RNA sorting process are being actively explored. Research identified molecules such as neural sphingomyelinase 2 (nSMase2) [15], AGO2 protein [32], RNA-binding proteins (in particular Major Vault Protein) [35], 3′end nucleotide additions of RNAs [36], or endogenous RNAs [37] as mediators influencing RNA sorting. A recent summary of miRNA loading mechanisms is reviewed in [38]. In addition, lipid raft regions in the membrane of MVB are suggested to be involved in the process of RNA incorporation into exosomes. A selective lipid-mediated mechanism of RNA loading into exosomes has been proposed [28]. In this model, binding of an RNA molecule to the raft-like region occurs before the budding-in process of the MVB membrane, and RNA is delivered to the outer (cytoplasmic) surface of the MVB limiting membrane by RBPs (RNA-binding proteins) [39,40,41]. As previously suggested [42], there can be a few different ways of RNA binding to the membrane of MVB: (1) RNA may bind directly to the membrane raft region and dissociate from RBPs, (2) RNA may bind directly to the membrane rafts and remain bound to RBPs, or (3) RNA may be attached to the membrane surface via RBP binding to the raft region. Other studies report that the presence of specific RNA motifs such as EXO-motifs [43] and RAFT-motifs [42] in the sequence of RNA may regulate the binding of RNA aptamers to the lipid raft regions of the membrane.

RNA motifs are short sequences in the RNA related to a particular property, such as binding to certain molecules and sorting into exosomes. RNA motifs that are significantly over-represented in exosomes can be designated as EXO-motifs [43]. They are more abundant in exosomal RNA than in cellular RNA. Researchers report the identification of various EXO-motifs within isolated exosomal RNAs derived from exosomes produced by various cells such as T lymphoblasts [43], herpesvirus-infected lymphoma cells [44], and hepatocytes [45]. EXO-motifs were also present within exosomal miRNA sequences derived from cancer cells [46]. RNA motifs that are significantly over-represented in RNAs bound to membrane lipid rafts can be designated as RAFT-motifs (raft-binding motifs) [42]. These recently identified RAFT-motifs were found to be most frequent both in raft RNA aptamers and exosomal pro-tumoral miRNAs that are transferred within exosomes from cancer cells to innate immune cells such as macrophages, natural killer cells, and dendric cells [42].

Application of exosomes as an RNA-delivery system is based on isolation of exosomes from extracellular fluids with subsequent loading of exosomes in vitro with appropriate RNA. The methodology proposed in this study is based on the loading of cells with appropriate RNA (fRNAa, functional RNA aptamer containing both RAFT-motif and a therapeutic motif) and allowing the cell to load fRNAa into ILVs with subsequent release of fRNAa within exosomes into the extracellular fluid. However, no clear relationship between the affinity of RAFT-motifs (CCCU, UCCC, CUCC, UUGU) to membranes and their location within the secondary or tertiary RNA structure was demonstrated, therefore, RAFT-motifs seem to function as sequence motifs rather than structural motifs.

Since the selective binding of RNAs to the membrane of MVB may be regulated by the activity of a lipid raft region in the MVB membrane and the presence of the RNA motifs, in this study we investigate the significance of these factors (i.e., lipid rafts and RNA motifs) in the RNA-membrane relationship. We analyze the interactions of certain RNA oligonucleotides containing EXO-motifs and/or RAFT-motifs with both ordered and disordered regions of membranes using model membranes. The aim is to determine which of these RNAs binds best to membranes and what is the role of RNA motifs in that process. We also explore the importance of the location of certain EXO- or RAFT-motifs within the secondary structure RNA molecules for their interaction with the raft membrane.

## 2. Results

The experimental system was designed to characterize the interaction of individual RNA aptamers containing different RNA motifs with both ordered and disordered membranes. We used liposomes made of DOPC, sphingomyelin, and cholesterol (molar ratio 6:3:1) or solely DOPC liposomes as models of ordered and disordered membranes, respectively. The fluorescent methods used were focused on the FRET technique applied to RNA aptamer-to-membrane energy transfer. The decrease in the fluorescence in the donor channel (YOYO-RNA) at a constant concentration of the donor and increasing concentration of the acceptor (LissRh-PE-Liposomes) was measured. Based on fluorescent measurements the dissociation constant (K_D_) was calculated, showing the degree of the RNA aptamers’ affinity to the lipid bilayers.

### 2.1. Motif Profile of RNA Aptamers

To check whether there are EXO- and/or RAFT-motifs in the random RNA pool, and where in the RNA secondary structure they are likely to be located, a search for RNA motifs was performed. The original RNA pool (148 oligonucleotides) was searched for EXO-motifs (GGAG, UGAG, CCCU, UCCU) [43] and RAFT-motifs (CCCU, UCCC, CUCC, UUGU) [42] (Table 1). Interestingly, the CCCU sequence seems to be a common RNA motif with identified affinity for both exosomes and raft membranes. In the original pool of 148 oligonucleotides, the CCCU motif was most frequent (20% occurrence) while the GGAG was the least frequent (2%) (Table 1). Other EXO-motifs, UGAG and UCCU, had 6% and 19% frequency, respectively, whereas RAFT-motifs occurred with 16% to 19% frequency.

For the measurements of the RNA-membrane interactions, ten RNA aptamers containing RNA motifs in various sequence locations were chosen (Table 1 and Table 2). Similar to the initial RNA pool, the CCCU motif was the most frequent sequence in this selected group of oligonucleotides. Regarding this motif, four RNA aptamers have a single CCCU motif (RNA number 10, 13, 64, and 93), three RNAs have double CCCU motifs (aptamer 19, 24, and 102) and finally, RNA 54 contains three CCCU motifs. As a comparison, two RNAs with no CCCU motif were chosen: RNA 90 has one single EXO-motif and a single RAFT-motif different than CCCU (motif GGAG) and RNA 78 has one EXO-motif and no RAFT-motifs at all. Other EXO and RAFT-motifs for each aptamer are listed in Table 2.

The RNAs’ secondary structures were predicted using the m-fold program [47] and the possible location of RNA motifs was marked (examples in Table 2). RNA motifs were in general located in the same stem/loop arrangement over alternative aptamer structures predicted by m-fold (Supplementary Table S1). The difference in the free energy between the first and second predicted structure varied from 0.1 to 1.3 kcal/mol, with an average difference of 0.5 kcal/mol.

### 2.2. Binding of RNA Aptamers to the Ordered and Disordered Lipid Membranes

**Figure 1:**
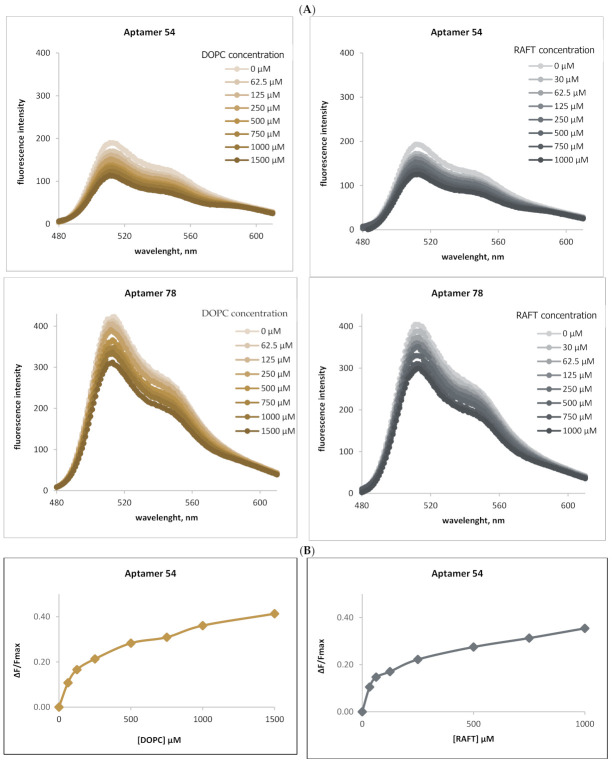

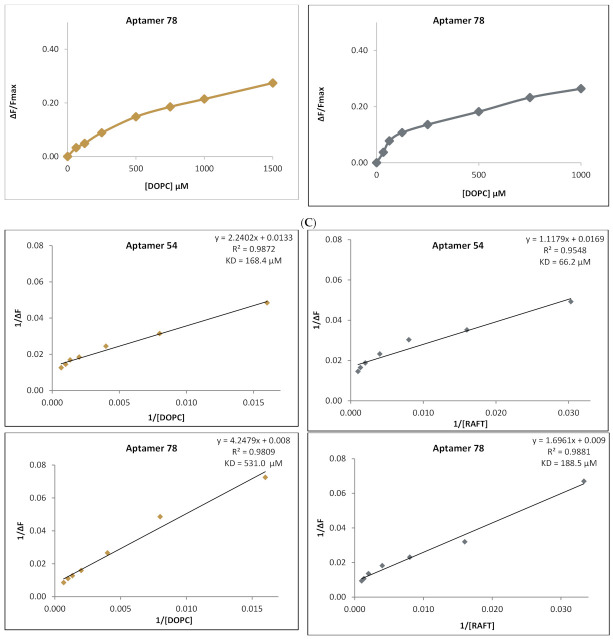
The interactions of YOYO-1-labeled RNA aptamers 54 and 78 with DOPC or RAFT liposomes. (**A**) Emission spectra of aptamer bound YOYO-1 (excitation at 466 nm), titrated with DOPC liposomes (0 µM–1500 µM) (left) and RAFT liposomes (0 µM–1000 µM) (right). The fluorescence intensity of YOYO-1 decreases with the increase in liposome concentration, for both aptamers 54 (top chart) and aptamer 78 (bottom) indicating the energy transfer to the membrane-bound acceptor (Liss Rhod PE). RNA aptamer concentration 0.25 µM, YOYO concentration 2.5 µM. (**B**) Change of YOYO-1 fluorescence during titration of RNA aptamer 54 (top) and RNA aptamer 78 (bottom) with DOPC liposomes (left) or RAFT liposomes (right). F_max_ is the maximal YOYO-1 fluorescence obtained before acceptor-liposome titration. ΔF is the difference between measured fluorescence and F_max_ (**C**). Reciprocal of YOYO-1 fluorescence change during titration with DOPC (in left) or RAFT (right) liposomes. The calculated K_D_ values indicate stronger RNA aptamer interaction with RAFT liposomes. For RNA aptamer 54—membrane measurements (top), the K_D_ values are 168.4 µM and 66.2 µM with the interaction with DOPC and RAFT liposomes, respectively; for RNA aptamer 78, the K_D_ values are 530.0 µM and 188.5 µM for interaction with DOPC and RAFT liposomes, respectively. The regression line equation and the coefficient of determination (R^2^) are noted above the calculated K_D_ value in the top-right corner of the chart.

To confirm the importance of lipid rafts in RNA-membrane interactions, fluorescence measurements were applied based on the FRET mechanism. For each selected RNA aptamer, the changes in donor emission intensity were observed during titration with disordered (DOPC) liposomal membranes or with liposomal membranes containing ordered domains (DOPC, SM, CHL). Figure 1 shows an example of this approach, illustrating the interactions of YOYO-1- labeled RNA aptamer 54 (RNA 54) and 78 (RNA 78) with both ordered and disordered membranes. Results for the other raft-RNA aptamers are presented in the Supplementary Materials (Figure S1).

As shown on the emission spectra (Figure 1A) for RNA aptamer 54 (top) and RNA aptamer 78 (bottom), a decrease in RNA-bound YOYO-1 fluorescence was observed during titration with disordered DOPC liposomes (left) and ordered RAFT liposomes (right) containing ordered domains. This indicates the energy transfer from donor RNA-bound YOYO-1 to the membrane-bound acceptor LissRh-PE. In Figure 1B the change of YOYO-1 fluorescence during titration with DOPC (left) and RAFT (right) liposomes is shown. F_max_ is the maximal YOYO-1 fluorescence obtained before liposome titration. ΔF is the difference between F_max_ and subsequent fluorescence measurements. The ratio of ΔF/F_max_ increases during titration of both types of liposomes, showing the gradual saturation of RNA with lipid molecules.

The linear regression of the aptamer-bound YOYO-1 fluorescence changes during titration with DOPC (left) and RAFT liposomes (right) is presented in Figure 1C. The resulting reciprocal lines were used to calculate the dissociation constants (K_D_). The calculated K_D_ values were higher for the interaction of RNA aptamers with DOPC liposomes in comparison with RAFT liposomes. For aptamer 54, the K_D_ was 168.4 µM for the interaction with DOPC and 66.2 µM for interaction with RAFT liposomes (Figure 1C). Similarly, for RNA aptamer 78 the K_D_ values were 530.0 µM and 188.5 µM for the interaction with DOPC and RAFT liposomes, respectively. Similar differences in the K_D_ values were observed for the other tested RNA aptamers interacting with ordered and disordered membranes (Supplementary Figure S1).\

**Figure 2:**
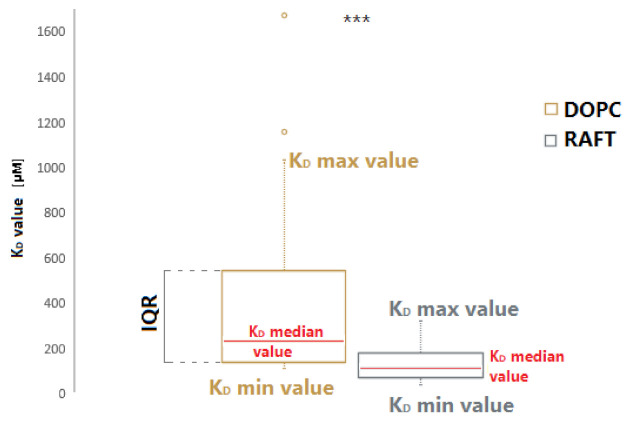
The range of K_D_ values for binding RNA aptamers to DOPC and raft membranes. The box shows the interquartile range (IQR) of the K_D_ measurements. The comparison of the median K_D_ values (marked in red) for binding RNA aptamers to DOPC membranes is 232 µM (left) and to raft membranes is 113 µM (right), indicates stronger RNA aptamer interaction with raft membranes rather than disordered membranes. The whiskers (dotted line) restrict the K_D_ value range and mark the minimal and maximal K_D_ values. The circles in the top of the boxplot are the outliers (n_DOPC_ = 20, n_RAFT_ = 30). The symbol *** means that *p* < 0.001 (Mann–Whitney U test of differences in medians).

The boxplot of the K_D_ values range is presented in Figure 2 for both DOPC and RAFT membranes. The median dissociation constant (K_D_) value was over two times lower for RNA-RAFT interactions than the median K_D_ value for RNA-non-RAFT liposomes. The comparison of median RNA-DOPC K_D_ value (232 µM) and median RNA-RAFT K_D_ value (113 µM) indicates that the presence of membrane rafts promotes the binding of RNA aptamers to the liposome membranes. A significant difference was also confirmed by the Mann–Whitney U test (U = 112, *p* < 0.001).

### 2.3. Analysis of EXO- and RAFT-Motifs within RNA Aptamers

**Figure 3:**
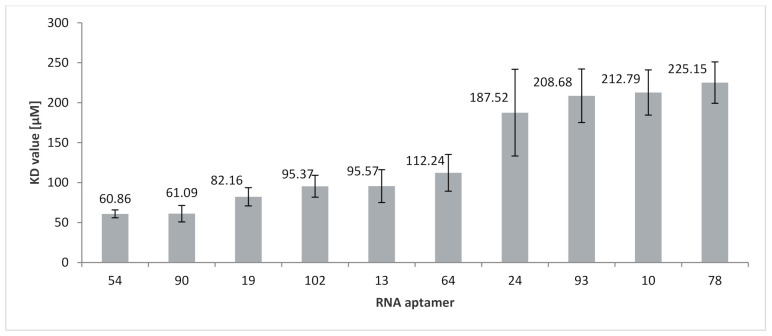
Comparison of the K_D_ values for binding of RNA aptamers to RAFT liposomes. The K_D_ values are the mean ± SE of 3–4 independent experiments.

To determine the importance of RNA motifs (both EXO and RAFT) for RNA affinity to membranes, the K_D_ values were compared (Figure 3; Table 2). The strongest affinity, characterized by the lowest K_D_ value (60.9 µM), was obtained for RNA 54 which contains three CCCU motifs (two of which are located entirely in loops). Another EXO-motif, UCCU, also occurs three times in this RNA and within the loop domain. Besides CCCU, three other RAFT-motifs are present in this aptamer: UCCC, CUCC, and UUGU. The two last motifs are present twice in this RNA. These RAFT-motifs share their location between the stem and loop domain. It should be noted that three groups of motifs overlap: UCCU with UUGU, UCCU with CUCC, as well as CUCC, UCCC, and CCCU. The second highest affinity (K_D_ = 61.1 µM) was obtained for the aptamer 90, which has an EXO-motif GGAG and a RAFT-motif UUGU.

The highest K_D_ value is observed for RNA 78 with just one EXO-motif (UGAG) and no RAFT-motifs. In contrast, the lowest affinity for lipid rafts is measured for RNA 78 (K_D_ = 225.2 µM) which, besides EXO-motif UGAG, has no other motifs. The UGAG motif is located partially in the loop and stem sections. The other eight aptamers have K_D_ values in the range of 61 to 213 µM. Besides RNA 54, four other aptamers (RNA 90, 19, 102, and 13) have a K_D_ value below 100 µM, and RNA 64, 24, 93, and 10, as well as RNA 78, have K_D_ values above 100 µM, wherein a significant increase in the K_D_ value is observed between RNA 64 (K_D_ = 112.2 µM) and RNA 24 (K_D_ = 187.5 µM).

The K_D_ value varies among aptamers with a different number of CCCU motifs. The average K_D_ value for aptamers with a single CCCU motif (RNA 10, 13, 64, and 93) is equal to 139 µM (±29) and decreases to 121 µM (±27) for aptamers with two CCCU motifs (RNA 19, 24, and 102). These findings, in conjunction with the K_D_ value of aptamer 54 with three CCCU motifs (K_D_ = 61 µM), may indicate a relationship between the number of CCCU motifs and RNA-membrane affinity, although the strong affinity of aptamer 90 (no CCCU motif) suggests that other raft-binding motifs, such as UUGU, also enhance the RNA affinity to membrane rafts.

To this point, we have documented the importance of both lipid rafts and RAFT-motifs for RNA-membrane interactions. We now wish to show that several raft-RNA aptamers [42] contain both miRNA- and raft-binding motifs. Bioinformatics analysis revealed three—and twenty-one—human miRNAs with a 12-nts and 11-nts, respectively, reversed/complement motif to raft-RNA aptamers (Table 3).

**Figure 4:**
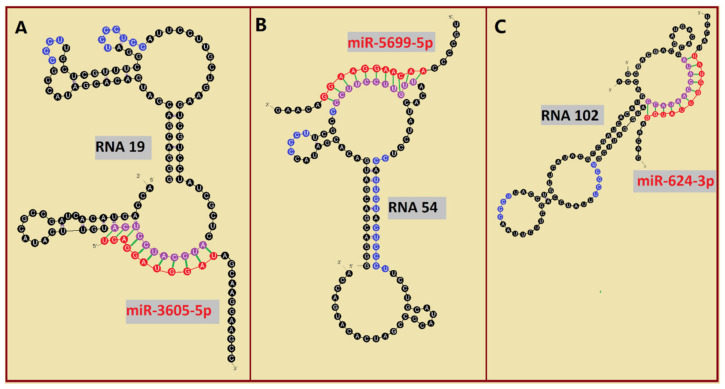
Binding of human miRNAs to bifunctional RNA aptamers containing both raft- and miRNA-binding motifs. (**A**) RNA 19 interacting with miR-3605-5p. (**B**) RNA 54 interacting with miR-5699-5p. (**C**) RNA 102 interacting with miR-624-3p. The RNA aptamer’s raft-binding motifs are marked in blue, and the miRNA-binding motif is marked in purple. The miRNA reversed/complement motif is marked in red.

Among analyzed raft-RNA aptamers, there are a few special cases: RNA aptamers containing two different miRNA-binding motifs for two different miRNAs (e.g., RNA 10, RNA 14), RNA aptamers containing the same miRNA-binding motifs for two different miRNAs (e.g., RNA 157), two different RNA aptamers containing two different miRNA-binding motifs for one miRNA (e.g., RNA 49, RNA 67). Interestingly, 5-nts raft-RNA motifs UCCCU and CUCCC (an overlap of CUCC, UCCC, and CCCU raft-RNA motifs) appear to be the most frequent motif within miRNA-targeting raft-RNA aptamers. Three raft-RNA aptamers (RNA 19, RNA 54, RNA102) contain a miRNA-binding motif and all have a strong affinity to rafts (Table 2). Schematic representation of miRNAs binding to these raft-RNA aptamers is shown in Figure 4. Table 3 and Figure 4 show examples of fRNAa with binding motifs to both lipid rafts and human miRNAs.

### 2.4. Analysis of RNA Aptamers Affinity to Exosomal Membranes

**Figure 5:**
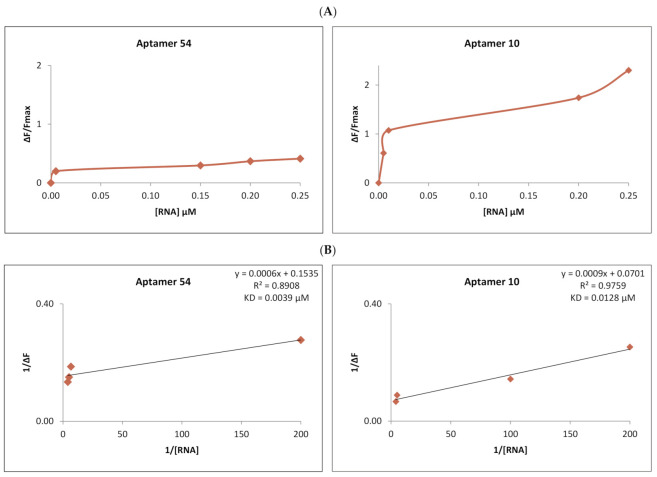
Comparison of the interaction of the strongest binding aptamer, RNA 54 (left), and the least strong, RNA 10 (right), to purified exosomes. (**A**) Change in the fluorescence of exosome-bound CTB-555 and fDiI during titration with RNA aptamer 54 (left) and RNA aptamer 11 (right). F_max_ is the maximal CTB-555 and fDiI fluorescence obtained before titration with RNA acceptor. ΔF is the difference between measured fluorescence and F_max_. (**B**) Reciprocal of CTB-555 and fDiI fluorescence change during titration with RNA aptamer 54 (in left) and aptamer 10 (right). The regression line equation and the coefficient of determination (R^2^) are noted above the calculated K_D_ value in the top-right corner of the chart.

We have also determined the raft-RNA aptamers’ activity in biological systems using exosomes. After purification and labeling (Section 4), exosomes were exposed to the activity of the different RNA aptamers. As previously for RNA-liposome interactions, also here the FRET measurements were taken. The decrease in the fluorescence in the donor channel (CTB-555 and FAST DiI—labeled exosomes) was measured under an increasing concentration of the acceptor (SYTO 61-labeled RNA). The dissociation constant (K_D_) was calculated for the RNA-exosome complex. Figure 5 compares two examples of these exosome-RNA interactions. The strongest RNA-to-exosome binding was obtained for aptamer 54 with the K_D_ = 0.0039 µM, whereas the least strong aptamer was RNA 10 (K_D_ = 0.0109 µM). The results of the remaining RNA aptamers are attached in the Supplementary Materials (Figure S2).

**Figure 6:**
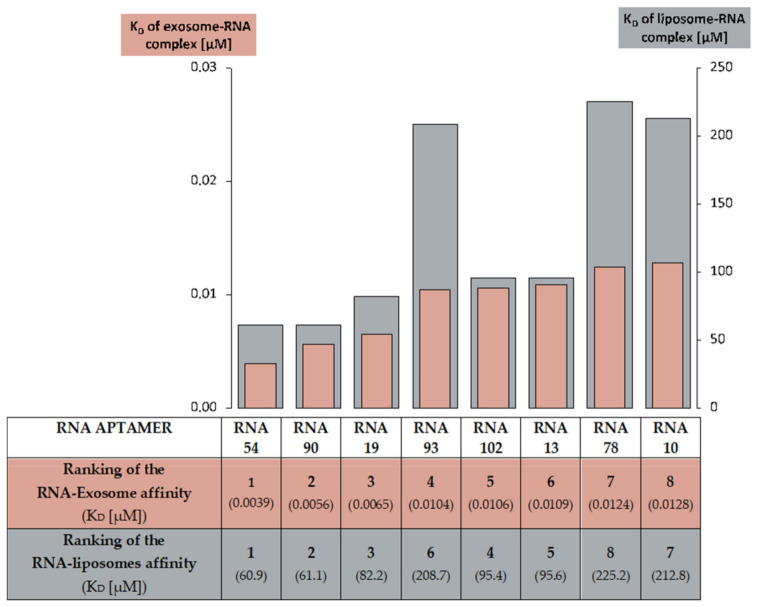
A comparison of the RNA aptamer affinity to raft liposomes and to exosomes. The ranking of RNA-to-raft liposomes affinity was based on the RNA-liposome complex K_D_ calculations. The ranking of RNA-to-exosomes affinity was based on K_D_ for the RNA-exosome complex.

The differences in affinity of eight analyzed RNA aptamers to exosomes, seem to resemble the variations of these same RNA aptamers’ relationship found in the liposome-RNA assay (Figure 6). In both experiments, RNAs 54, 90, and 19 had a strong affinity for membranes, while RNAs 10 and 78 expressed the least strong RNA-membrane relationship.

## 3. Discussion

### 3.1. The Importance of Lipid Rafts for Binding RNA Aptamers to Membranes

In our study, the affinities of lipid membranes to RNA oligonucleotides containing both EXO- and RAFT-motifs were characterized to understand the role of RNA sequence and membrane lipid rafts on the aptamer-membrane interactions. Using the FRET approach, the affinity of RNA aptamers to lipid membranes was measured, while analyses of the secondary structure of the RNA provided data about the location of the RNA motifs within the RNA molecule.

To confirm the role of lipid rafts in RNA-membrane interactions, fluorescence measurements were performed. For each of the individual RNA aptamers, the changes in donor (YOYO-1) emission intensity were measured after incubation with ordered RAFT (DOPC:SM:CHL) and disordered non-RAFT (DOPC) membranes. All RNA aptamers showed a higher affinity for RAFT liposomes compared to non-RAFT liposomes. The dissociation constant (K_D_) values were approximately two times lower for the RNA-RAFT interactions than for the RNA interaction with non-RAFT liposomes (Figure 2). These results indicate that the presence of membrane rafts promotes the binding of RNA aptamers to the liposome membranes. We have also confirmed these results through the determination of the dissociation constant values of exosome-RNA aptamer complexes.

Similarly, stronger RNA affinity to raft membrane was obtained in the study on the interactions of the *VegT* mRNA localization signal to membranes in *Xenopus* oocytes [48]. The VegT212 RNA (RNAs that localize to the vegetal pole during oogenesis) bound strongly (K_D_ value ~27 times lower) to the liquid-ordered bilayer in comparison to the liquid-disordered (DOPC) bilayer, and the localization elements were suggested to function as novel lipid raft-binding RNA motifs. The presence of localization elements within the loop of an RNA hairpin structure enhanced RNA affinity for lipid rafts.

The structure-dependent RNA binding was demonstrated previously for membranes in the liquid-disordered, liquid-ordered phase, and gel phase [49]. Although the studies did not identify binding motifs, they showed that there is a difference in the specificity of RNA binding to rafts depending on the RNA structure. A recent study [50] indicates that while most of the randomized RNAs in the mixture of RNA sequences may bind to the gel membranes (L_β_-maximally ordered phase), there is a rather small amount of RNAs with the affinity for fluid membranes (L_α_-liquid disordered phase). These studies emphasize that the state of the membrane order can regulate the RNA-membrane interactions and RNAs can moderately interact with the raft phase.

Lipid rafts were reported to be involved in the vesicle formation process itself [51] and it was proposed that lipid rafts participate in selective RNA incorporation into exosomes [28]. The presence of a raft-like region in the membrane of MVB seems to allow a direct interaction of RNAs with this raft-like region [28] and/or the interaction of the RBP–RNA complex with the raft region through the RBP [14]. Ceramide molecules present within the MVB membrane can induce a coalescence of microscopic rafts into a large-membrane macrodomain which in turn can induce a budding-in process and formation of ILVs [28]. Interestingly, the preferential binding to lipid-raft microdomains was reported for a broad range of molecules under both physiological and pathological conditions such as polysialic acid [52], human amyloid-β peptide [53], protooncogenes such as N-ras protein [54], the Shiga toxin [55], as well as virus glycoproteins [56]. Thus, the role of raft domains as platforms for interactions of various molecules with lipid membranes appears to be significant. Our results are consistent with this function of lipid rafts and indicate a role of these liquid-ordered domains in the process of RNA loading into exosomes.

### 3.2. The Importance of EXO- and RAFT-Motifs in Binding of RNA Molecules to Membranes

Since the presence of specific RNA motifs in the sequence of RNA is postulated to regulate the binding of RNAs to the lipid raft regions of the membrane, we searched the original RNA pool for EXO- motifs (GGAG, UGAG, CCCU, UCCU) [43] and RAFT-motifs (CCCU, UCCC, CUCC, UUGU) [42]. Interactions between individual RNA aptamers containing various EXO- and RAFT-motifs with membrane lipid rafts were analyzed, and the dissociation constant (K_D_) values of aptamer-membrane affinity were compared. RNA aptamers containing both the EXO- and RAFT-motif CCCU bound stronger to lipid rafts compared to the control RNA aptamer without the RAFT-motif. RNA aptamer 54 with three CCCU motifs (two of them in loops) exhibited the highest affinity for the raft bilayer, suggesting that the number and location of RNA motifs may be meaningful in RNA binding to the membrane.

Our results support the concept in the study [42] where RAFT-motifs were suggested to be involved in the mechanism of direct binding of miRNA to the raft region of the MVB, as well as the aforementioned study reporting that the presence of RNA localization elements within the loop of the *VegT* RNA structure enhances its binding to lipid raft domains [48] (although in the current research, a special meaning of the hairpin structure could not be determined).

As for the EXO-motifs, our results confirm, that some of these sequences such as the CCCU motif may similarly interact with membranes directly. However, others such as UGAG (a GGAG derivate), did not enhance the affinity of the RNA aptamer to the lipid membranes. Interestingly, regarding the second best-binding aptamer (RNA 90) (K_D_ = 61.1 µM), its affinity may not be caused by the GGAG motif. The sequence AGUG, present within a loop of RNA 90, is overlapped with the EXO motif GGAG. Analysis of the RNA 90 structure revealed complementary antiparallel sequences: AGUG (nucleotides 28–31) and CACU (nucleotides 57–60). The CACU sequence is partially within a bulge of RNA 90, therefore these leftward tetranucleotide loop/bulge sequences could efficiently hybridize leading to the formation of a “kissing loop/bulge complex” [57], thus inactivating the GGAG motif. In addition, the RAFT-motif UUGU, present partially within the bulge of RNA 90, seems to be the strongest RAFT-motif based on the correlation analysis of 4-nts motifs from two sets of RNAs: exosomal pro-tumoral miRNAs and raft RNA aptamers [42]. Therefore, the RAFT-motif UUGU (not the EXO motif GGAG) is responsible for the strong affinity of RNA 90 to the lipid raft region. It may be that the GGAG motif and its UGAG derivate do not enhance direct RNA chain—membrane interactions, but rather are involved in the RNA chins interactions with membranes mediated by RBPs. The GGAG sequence was reported to have an affinity to RBP (hnRNPA2B1) and prion protein (PrP) [43,58]. In the research performed by Cha et al., the GGAG motif was found in the members of the miR-320 family [59], but not found in other studied exosomal miRNAs. Although in that study no general enrichment for specific RNA motifs was found, researchers suggested that individual miRNAs might undergo sequence-specific sorting into exosomes. It was also observed that pre-miR-181a-1 molecules were associated with the outer surface of late endosomes/lysosomes in both axons and growth cones suggesting that pre-miRNAs are docked to the outer membrane surface of these vesicles for transport [60]. Interestingly, both pre-miR-181a-1 and miR-181a-3p contain a RAFT-motif UUGU thus suggesting the involvement of this RAFT-motif in the docking process of pre-miR-181a-1and miR-181a-3p molecules to membranes.

The present study confirms the importance of RAFT-motifs as well as some EXO-motif in facilitating RNA binding to membrane rafts. A particular CCCU motif, which is common for both EXO- and RAFT-motifs, appeared to enhance RNA aptamer-raft interactions. The loop location of the motif seems to play a role in enabling the accessibility of the motif for the interaction with the raft lipids. Thus, our study supports the concept that the RNA sorting process into exosomes is highly dependent on the presence of these motifs and raft-like regions within the MVB membrane.

### 3.3. Potential Medical Applications of Functional RNA Aptamers (fRNAa)

RNA aptamers are single-stranded RNA oligonucleotides with the ability to bind to target molecules with high affinity and specificity. They are generated through a selection process, the systematic evolution of ligands by exponential enrichment (SELEX) [61,62]. Selected aptamers are extensively studied for diagnostic and therapeutic applications [63,64]. They were reported to be a promising therapeutic agent in such conditions as neurodegenerative diseases [58,65,66] and cancer [67,68]. Moreover, interfering RNAs (iRNA) such as siRNA or miRNA are being investigated for specific gene silencing in the treatment of various diseases [69,70,71]. In 2018, patisiran (Onpattro; Alnylam Pharmaceuticals, Cambridge, MA, USA), the first RNAi drug, was approved by the US Food and Drug Administration (FDA) for the treatment of hereditary transthyretin amyloidosis (hATTR) with polyneuropathy [71,72]. This gene silencing drug carries siRNA that inhibits the production of an abnormal form of transthyretin in the liver.

In studying these oligonucleotide therapeutics, researchers simultaneously work to overcome challenges that occur in treatments such as non-specific delivery of therapeutic RNAs, stimulation of immunogenicity by delivered RNAs or its carriers, risk of degradation by extracellular nucleases, or finding a suitable vector to deliver the therapeutic agent [69,70,73]. One possible solution to that problem seems to be employing exosomes as carriers of therapeutic factors [64,74,75,76,77]. Exosomes were reported to effectively deliver therapeutic cargo for cancer treatment [70,77], neurodegenerative diseases [65,78] and virus infections including COVID-19 [79,80]. Interestingly, aptamers could also be used as ligands implemented to the surface of engineered exosomes hereby enhancing their targeting efficiency to the recipient cells [64,81].

**Figure 7:**
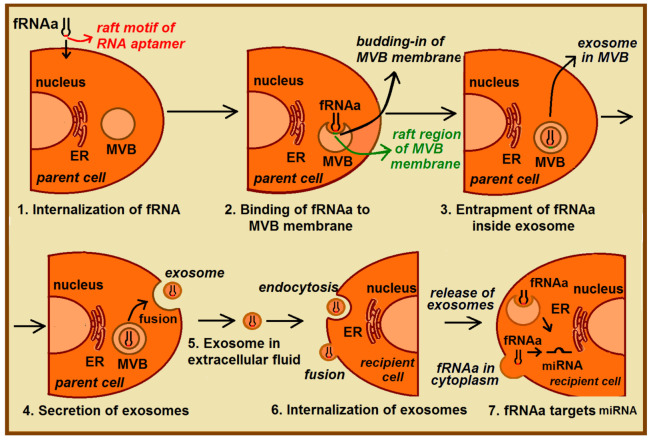
Proposed fRNAa delivery mediated by exosomes. The functional RNA aptamer (fRNAa) owns a therapeutic domain as well as RAFT-motif sequence and its delivery involves a few stages: (1) Internalization of the fRNAa by donor cells; (2) binding of fRNAa to the raft-like domains inside membrane of MVB; (3) entrapment of fRNAa inside ILV (exosomes), resulted from the budding-in process of MVB membrane; (4) secretion of exosomes by the fusion of MVB with the parent cell membrane; (5) exosomal transport of fRNAa in the extracellular fluids; (6) internalization of exosomes by recipient cell, resulted from the endocytosis or/and fusion of exosomes with plasma membrane; (7) release of exosome content, which allows fRNAa reach the target miRNA and induce desired effects inside the target cell.

Still, a direct sorting of therapeutic RNAs into exosomes presents a challenge due to the low efficiency of loading and delivery of therapeutic cargo to target cells [51]. In the proposed strategy of exosomal delivery of therapeutic RNAs, the functional RNA aptamer (fRNAa) is constructed (Figure 7). This functional construct, besides having a therapeutic motif (such as RNA-based anti-miRNA inhibitors, siRNA, or miRNA) specific against certain molecular targets (such as mRNA, miRNA) [82], also includes a RAFT-motif(s) inside the fRNAa sequence that facilitates its loading into ILV (exosomes). Table 3 and Figure 4 show examples of such bifunctional fRNAa with binding motifs to both lipid rafts and human miRNAs. The fRNAa are introduced into donor cells, where RAFT-motifs enhance binding of fRNAa to the limiting membrane of MVB and thus support a loading mechanism of therapeutic RNA into ILV. Released fRNAa is protected within exosomes. Upon reaching the target cells, exosomes are internalized by fusion of exosomal membrane with the plasma membrane or by endocytosis. The release of both fRNAa and miRNAs [83] from endosomes into cytoplasm can occur in a pH-dependent manner through the internal fusion of the exosomal and endosomal membranes, allowing the therapeutic domain activity. The proposed procedure does not require exosome isolation. In future studies, one can apply CRISPR/Cas9-based genome editing [82] for generating raft-motif containing anti-miRNA RNA inhibitors.

In the last years, the molecular mechanisms of exosome RNA sorting have been better understood, however, there are still gaps in its full understanding that limit the application of exosome-based therapies. A previously constructed bifunctional RNA aptamer with the affinity to both membranes and the amino acids (specific for tryptophan) [84] and a perspective of employing such bifunctional RNA aptamers containing regions with affinity to lipid rafts and regions with affinity to RBP [42], together with the results of this study, provides the basics for construction fRNAa, that may facilitate efficient loading of therapeutic RNA into exosomes. A better understanding of RNA-membrane raft interactions will allow further development of methods for selective RNA introduction into exosomes.

In conclusion, our results confirm the importance of both lipid rafts and RNA motifs for RNA-membrane interactions: (1) aptamers had a greater affinity for raft compared to non-raft membranes (2) the affinity of RNAs containing RAFT-motifs to RAFT liposomes was higher in comparison to an aptamer without these motifs. Results also suggest that the location of RNA motifs is important for the RNA-raft interactions. A potential application for personalized gene therapy is that the RAFT-motif can be incorporated into the sequences of therapeutic fRNAa, to enrich them in ILV during vesicle formation and after the vesicles are released from parent cells, exosomes can deliver fRNAa to target cells in vivo.

## 4. Materials and Methods

### 4.1. Materials

Lipids: 1,2-dioleoyl-sn-glycero-3-phosphocholine (DOPC); N-stearoyl-D-erythro-sphingosylphosphorylcholine (Stearoyl Sphingomyelin, SM); cholesterol (CHL) were purchased from Avanti Polar Lipids (Alabaster, AL, USA). Fluorescent probes: YOYO™-1 Iodide (YOYO-1, 1 mM Solution in DMSO), Lissamine Rhodamine B, 1,2-dihexadecanoyl-sn-glycero-3-phosphoethanolamine triethylammonium salt (LissRh-PE), Cholera Toxin subunit B (recombinant) Alexa Fluor 555 conjugate (CTB-555), SYTO 61, FAST DiI (fDiI), and calf bovine serum (CBS) were purchased from ThermoFisher Scientific (Waltham, MA, USA). T7 RNA polymerase was obtained from Epicentre (Madison, WI, USA). Sephacryl S-1000 superfine was purchased from Sigma-Aldrich (St. Louis, MO, USA). Millipore Ultrafree-MC Centrifugal Filter Devices with microporous membranes 100 nm pore size were purchased from VWR (Radnor, PA, USA).

### 4.2. Preparation of Large Unilamellar Vesicles (LUVs)

Two pools of vesicles (1) liposome RAFT consisting of DOPC, SM, CHL in molar ratio 6:3:1 and (2) plain DOPC liposomes were prepared by the thin-film hydration method followed by extrusion [85]. The appropriate lipids originally dissolved in chloroform/methanol (2:1) were mixed and the Lissamine Rhodamine B dye was added to both lipid solutions (final concentration Liss Rhod PE = 0.2 mol%) [52]. Solvents were evaporated under a stream of nitrogen gas. The pellet was next resuspended in warmed (60 °C) RNA buffer (50 mM HEPES pH 7.0, 50 mM NaCl, 5 mM MgCl_2_, 2 mM CaCl_2_,) and incubated in a thermoblock (60 °C) for 3 min. Multilamellar vesicles (MLVs) were formed by the handshaking technique until complete pellet dissolution. The suspension of MLVs has been treated by five freeze-thaw cycles (repeated immersion in liquid nitrogen, warming in a 60 °C thermoblock followed by vortex). Large unilamellar vesicles (LUVs) were prepared by extrusion using the Avanti MiniExtruder (Avanti Polar Lipids, Alabaster, AL, USA) with a filter pore diameter of 100 nm. The mean diameter of these LUVs is similar to the mean diameter of exosomes.

### 4.3. Preparation of RNA Aptamers

RNA aptamers were obtained using the SELEX method [65]. The initial RNA pool with 50-mer random region (50N) or 70-mer random region (70N) was generated by T7 polymerase by in vitro transcription of a DNA template of the sequence: 5′-TGG TCA TGT GAT CGG CGT ATG—50N or 70N—TAT CGT GTC ATC GTC GTC CCT ATA GTG AGT CGT ATT A-3′ (the underlined sequence is the T7-promoter region) [42]. The sequences of the selected RNA pool are listed in [42], Supplementary Materials, Table S1.

RNAs containing EXO- and/or RAFT-motifs were chosen from this original RNA pool for analysis. Individual RNA aptamers (brought to the concentration 2.75 µM in highly purified water) were folded by cooling from 65 °C to room temperature over 10 min, after addition of 10× RNA buffer, each time before the experimental series. The RNAs’ secondary structures were predicted using the m-fold program (http://www.unafold.org/mfold/applications/rna-folding-form.php accesed on 28 August 2021).

### 4.4. Fluorescent Measurements

RNA aptamer-to-membrane FRET (Forster Resonance Energy Transfer) between RNA-bound YOYO-1 (donor) and membrane-bound LissRh-PE (acceptor) was measured at room temperature by exciting YOYO-1 and monitoring the decrease in its emission intensity in the presence of FRET [52,84].

Each analyzed RNA after folding (2.5 µM in RNA buffer) was added to YOYO-1 solution in RNA buffer (YOYO-1 final concentration 2.5 µM, RNA 0.25 µM). The YOYO-RNA aptamer solution was titrated with RAFT- or DOPC-liposomes with incorporated LissRh-PE. RNA aptamers-to-membrane FRET was monitored as a decrease in donor (YOYO-1) emission intensity (λ_ex_ = 466 nm and λ_em_ = 511 nm) under increasing acceptor (membrane-bound LissRh-PE) concentration.

### 4.5. Determinaton of the Dissociation Constant K_D_ for RNA-Liposome Complex

Based on fluorescent measurements, the K_D_ constant (the dissociation constant) of the aptamer-membrane complex was calculated using the Langmuir isotherm equation. RNA aptamers binding data was subjected to a nonlinear, least-squares analysis using the Langmuir equation [49,52,56]: ΔF = ΔF_max_ [x/(K_D_ + x)] where: ΔF_max_ is the calculated maximal fluorescence change, x is the DOPC or RAFT lipid concentration, and K_D_ is the equilibrium dissociation constant for RNA-liposome complex. Liposomes without fluorescence probes were used to correct for light scattering.

### 4.6. Isolation and Labelling of Exosomes

Exosomes were isolated from calf bovine serum (CBS) as described [85,86]. Briefly, CBS was subjected first to 20-min centrifugation at 2000× *g*, and the supernatant was subjected to 18 h centrifugation at 126,000× *g*. The obtained pellet was washed with PBS and dissolved by gentle agitation, before re-pelleting it with ultracentrifugation at 126,000× *g* for 70 min, and next re-suspending in 120 μL PBS. This suspension was incubated with a membrane fluorescence probe CTB-555 (2 μg/mL, 30 min, 20 °C), followed by incubation with fDiI (1 μM, 15 min, 20 °C), and further purified using a Sephacryl S-1000 gel filtration column, followed by a 100-nm pore size ultrafiltration. The gel filtration column was eluted with RNA buffer. The gel filtration step removes both small lipoprotein aggregates and non-bound CTB-555 and fDiI.

### 4.7. Fluorescence Measurements and Determinaton of the Dissociation Constant K_D_ for RNA-Exosome Complex

Each analyzed RNA after folding (2.5 µM in RNA buffer) was added to SYTO-61 solution in RNA buffer (SYTO-61 final concentration 2.5 µM, RNA 0.25 µM). The exosome suspension was titrated with SYTO-61-RNA aptamer solution. Membrane-to-RNA aptamer FRET was monitored at room temperature as a decrease in donor (CTB-555 and fDiI) emission intensity (λex = 550 nm and λem = 570 nm) under increasing acceptor (RNA-bound SYTO 61) concentration. The K_D_ values were calculated in a similar way to the RNA aptamer-rafted liposome complex.

## Figures and Tables

**Table 1:** The occurrence of RNA motifs in the original RNA pool and in the pool of ten RNA aptamers analyzed for membrane binding.

Searched RNA Motifs		% of RNA Motif In Original RNA Pool (148 RNAs)	% of RNA Motif in Analysed RNAs (10 RNAs)
**both EXO- and RAFT-MOTIF**	**CCCU**	20%	29%
**EXO-MOTIFS**	**GGAG**	2%	4.5%
	**UGAG**	6%	4.5%
	**UCCU**	19%	18%
**RAFT-MOTIFS**	**UCCC**	19%	22%
	**CUCC**	16%	13%
	**UUGU**	18%	9%

**Table 2:** Analyzed RNA aptamers with marked location of RNA motifs within the secondary structure. Both EXO- and RAFT-motif CCCU is marked in green, other EXO-motifs (GGAG, UGAG, and UCCU) are marked in red and other RAFT-motifs (UCCC, CUCC, UUGU) are marked in blue. Abbreviations used to name the location of RNA motifs in the secondary structure of the RNA molecule: the number (from 1 to 4) refers to the number of nucleotides located in a particular position of the 4-nucleotide motif; L or S refers to the loop or stem location, respectively (e.g., 4L—means that all 4 nucleotides are located in the loop, 3S/1L—3 first nucleotides of the RNA motif are located in the stem, and the last one is located in the loop). The overlapping motifs are noted. The K_D_ values of aptamer-membrane raft interactions are the mean values (±SE) of three independent experiments. The RNA sequences were folded using the m-fold program [47] at 37 °C. The 2° structures presented have the lowest free energy among the structural variants predicted by m-fold. Locations of RNA motifs in the alternative structures are presented in the Supplementary Table S1.

RNA Name	RNA Length [nts]	RNA Nucleotides Composition	RNA 2° Structure	RNA Motifs (Location) both EXO- and RAFT-MOTIF EXO-MOTIFS RAFT-MOTIFS	K_D_ Value (±SE) [µM]
54	91	19 A’s 35 C’s 14 G’s 23 U’s	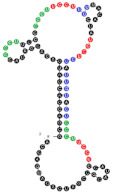	CCCU x3 (4L; 4L; 3S/1L) UCCU x3 (4L) UCCC x1 (3S/1L) CUCC x2 (3L/1S; 1L/3S) UUGU x2 (2L/1S/1L; 4S) overlaps: UCCU & UUGU UCCU & CUCC CUCC, UCCC & CCCU	60.9 (±5.0)
90	91	24 A’s 22 C’s 23 G’s 22 U’s	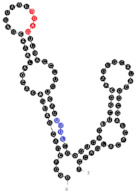	GGAG (3L/1S) UUGU (1S/1L/2S)	61.1 (±10.3)
19	111	22 A’s 39 C’s 21 G’s 29 U’s	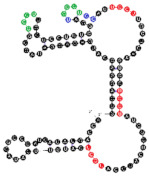	CCCU x2 (1S/3L; 3L/1S) UCCU x3 (4L; 4S; 3L/1S) UCCC (4L) CUCC (1L/3S) overlaps UCCC, CCCU & CUCC	82.2 (±11.4)
102	91	24 A’s 28 C’s 16 G’s 23 U’s	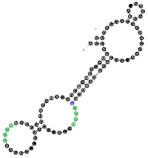	CCCU x2 (4L; 4L) UCCC (1S/3L) overlaps UCCC & CCCU	95.4 (±13.7)
13	91	20 A’s 23 C’s 23 G’s 25 U’s	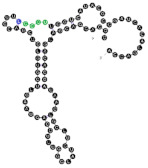	CCCU (2S/2L) UCCC (3S/1L) overlap	95.6 (±20.5)
64	90	21 A’s 25 C’s 20 G’s 24 U’s	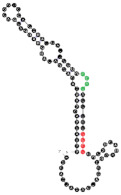	CCCU (3L/1S) UCCU (4S)	112.2 (±23.1)
24	111	23 A’s 33 C’s 28 G’s 27 U’s	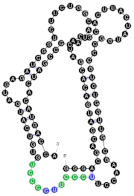	CCCU x2 (3S/1L; 4L) UGAG (3L/1S) UCCC x2 (4S; 4L) overlaps UCCC & CCCU 2x	187.5 (±54.3)
93	111	26 A’s 34 C’s 24 G’s 27 U’s	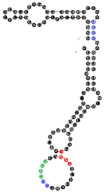	CCCU (4L) UCCU (2S/2L) UCCC x2 (4S; 4L) CUCC (4L) overlaps CUCC, UCCC & CCCU	208.7 (±35.5)
10	111	19 A’s 33 C’s 25 G’s 34 U’s	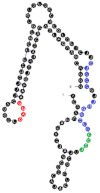	CCCU (4L) GGAG (1S/3L) UCCC x2 (4S; 4L) CUCC x2 (1L/3S; 1S/3L) UUGU (2L/2S) overlaps CUCC, UCCC & CCCU CUCC & UCCC	212.8 (±28.3)
78	91	21 A’s 26 C’s 23 G’s 21 U’s	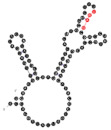	UGAG (1L/3S) No RAFT-motif	225.2 (±25.9)

**Table 3:** Raft-RNA aptamers containing both miRNA- and raft-binding motifs with corresponding human miRNAs containing reversed/complement motif.

Raft RNA Aptamer with miRNA- and Raft-Binding Motifs			Human miRNA with Reverse/Complement Motif	
Name	Human miRNA-Binding Motif	Raft-Binding Motifs	Name	Sequence, Reversed/Complement Motif in Red Color
RNA 60 RNA 103 RNA 113	12-nts long AUGCUCUCUGUA UGUCCAUUGUAU AGCCUUCUCCAU	CUCC UUGU, CUCC CUCC, UCCC, CCCU	miR-6818-5p miR-2355-5p miR-4531	UUGUGUGAGUACAGAGAGCAUC AUCCCCAGAUACAAUGGACAA AUGGAGAAGGCUUCUGA
RNA 9 RNA 10 RNA 10 RNA 14 RNA 14 RNA 19 RNA 32 RNA 41 RNA 43 RNA 48 RNA 49 RNA 53 RNA 54 RNA 67 RNA 68 RNA 102 RNA 108 RNA 119 RNA 140 RNA 142 RNA 153 RNA 157 **RNA 167** **RNA 167**	11-nts long CCUUCUCUCCA GUGAAAGGAGU UACUUUCUCUU GUAGUUACUUC CCCAGACCAGA AUCCAUCCUCA UCCCUCACUAG UCUGAAGGUUC AGAAUCUGCAU GCUCUUUUCAU GUUCGUCUUAU CCCCAGGACUC CCUUCCUUGUU CCUUUUGUUCG CUCCUCUGGCC AUACCAAUACC UUGUCCACCGC UCCCCUGGUCA ACUGAUCCAGC UCAUGCCCUCU CUUUUAGCCCU AUAUCAGCUCA UUCAGUUCUCA UUCAGUUCUCA	CUCC CUCC, UCCC, UUGU, CUCC, UCCC, CCCU CUCC, UCCC, UUGU, CUCC, UCCC, CCCU UCCC UCCC CCCU, UCCC, CCCU, CUCC UUGU, UCCC, CCCU UCCC, CCCU UUGU, CUCC, CCCU, CUCC, UCCC UUGU, UUGU, CUCC, UUGU CUCC, UCCC UCCC, CUCC, UCCC, CCCU CCCU, CCCU, UUGU, CUCC, UUGU, CUCC, UCCC, CCCU CCCU, UUGU CUCC, CUCC UCCC, CCCU, CCCU UUGU UCCC, CCCU, UUGU, UCCC, CCCU CUCC, CUCC, UCCC, UCCC CCCU, CUCC CCCU, UUGU, UUGU, CUCC CCCU, CUCC, CUCC, UCCC, CCCU CUCC, CCCU CUCC, CCCU	miR-7846-3p miR-4764-3p miR-3686 miR-548ao-5p miR-4786-3p miR-3605-5p miR-921 miR-3142 miR-5683 miR-3618 miR-208b-3p miR-3198 miR-5699-5p miR-208b-3p miR-4726-5p miR-624-3p miR-4638-5p miR-4465 miR-1287-5p miR-8052 miR-135b-3p miR-24-1-5p **miR-146b-5p** **miR-146a-5p**	CAGCGGAGCCUGGAGAGAAGG UUAACUCCUUUCACACCCAUGG AUCUGUAAGAGAAAGUAAAUGA AGAAGUAACUACGGUUUUUGCA UGAAGCCAGCUCUGGUCUGGGC UGAGGAUGGAUAGCAAGGAAGCC CUAGUGAGGGACAGAACCAGGAUUC AAGGCCUUUCUGAACCUUCAGA UACAGAUGCAGAUUCUCUGACUUC UGUCUACAUUAAUGAAAAGAGC AUAAGACGAACAAAAGGUUUGU GUGGAGUCCUGGGGAAUGGAGA UGCCCCAACAAGGAAGGACAAG AUAAGACGAACAAAAGGUUUGU AGGGCCAGAGGAGCCUGGAGUGG CACAAGGUAUUGGUAUUACCU ACUCGGCUGCGGUGGACAAGU CUCAAGUAGUCUGACCAGGGGA UGCUGGAUCAGUGGUUCGAGUC CGGGACUGUAGAGGGCAUGAGC AUGUAGGGCUAAAAGCCAUGGG UGCCUACUGAGCUGAUAUCAGU UGAGAACUGAAUUCCAUAGGCUG UGAGAACUGAAUUCCAUGGGU

## Supplementary Materials

The following are available online at www.mdpi.com/article/10.3390/ijms22179416/s1.
