# Supplementary material for: Role of RNA Motifs in RNA Interaction with Membrane Lipid Rafts: Implications for Therapeutic Applications of Exosomal RNAs
Source: Int J Mol Sci. 2021 Aug 30;22(17):9416. doi: 10.3390/ijms22179416 (PMC8431113; doi:10.3390/ijms22179416)
Supplement: Supplementary file 1 [file ijms-22-09416-s001.zip › ijms-1297205-Supplementary material/Figure S1.pdf]

**Figure S1. The interactions of other YOYO-1- labelled RNA aptamers with DOPC or RAFT liposomes.**

**A**. Emission spectra of aptamer bound YOYO-1 (excitation at 466 nm), titrated with DOPC liposomes (0 µM–1500 µM) (left) and RAFT liposomes (0 µM – 1000 µM) (right).

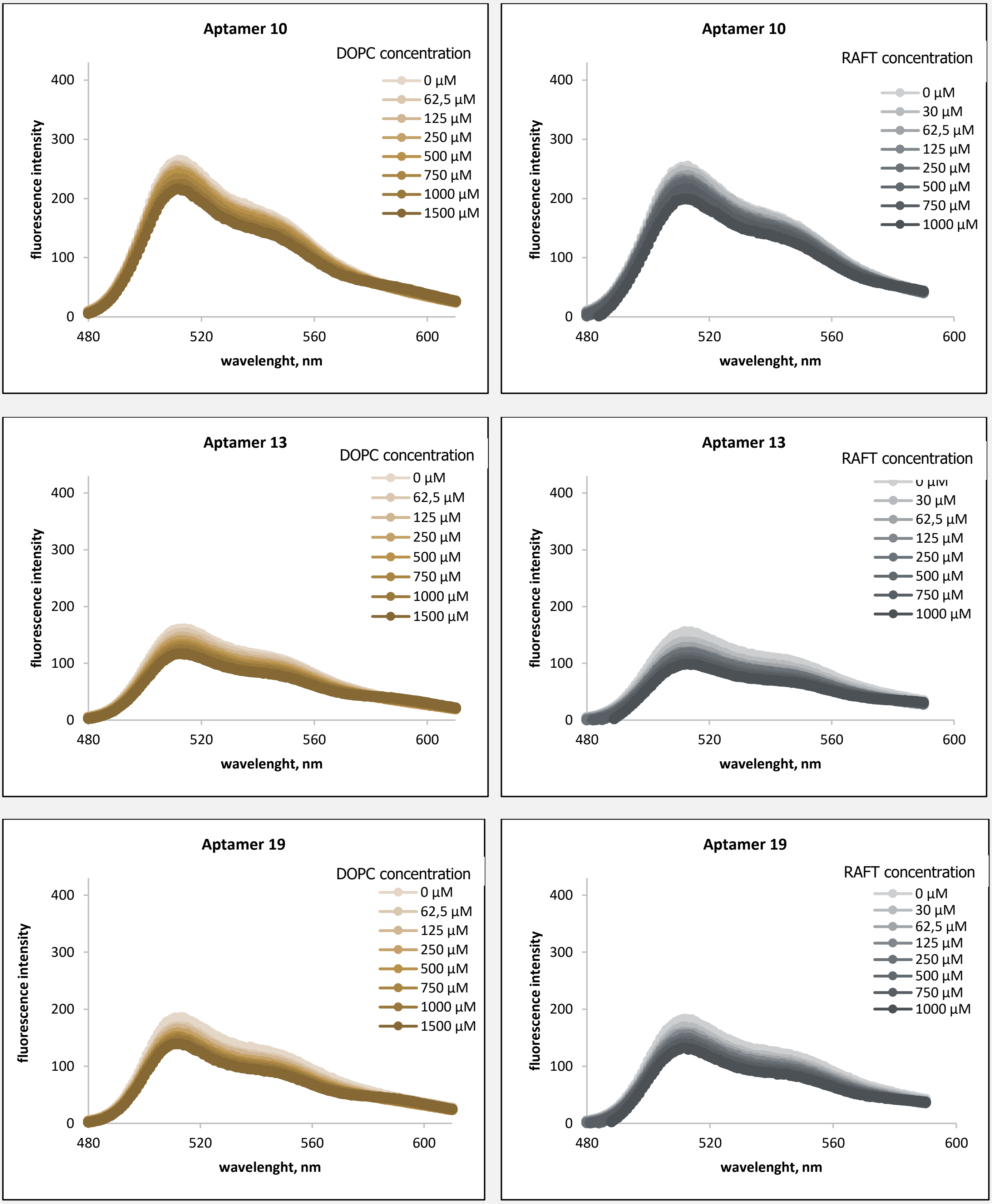

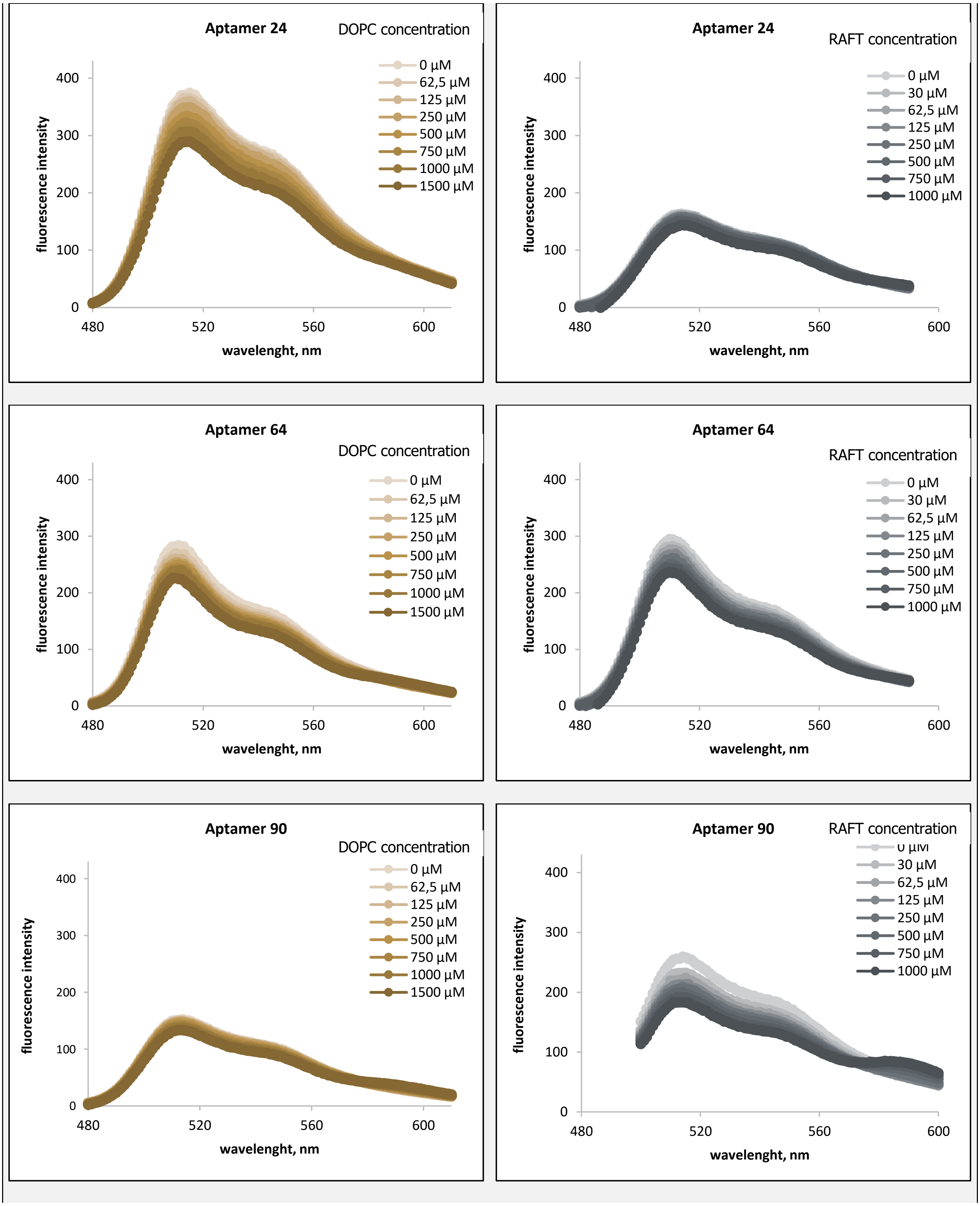
Aptamer 24
DOPC concentration
0 µM
62,5 µM
125 µM
250 µM
500 µM
750 µM
1000 µM
1500 µM
fluorescence intensity
wavelenght, nm
Aptamer 24
RAFT concentration
0 µM
30 µM
62,5 µM
125 µM
250 µM
500 µM
750 µM
1000 µM
fluorescence intensity
wavelenght, nm
Aptamer 64
DOPC concentration
0 µM
62,5 µM
125 µM
250 µM
500 µM
750 µM
1000 µM
1500 µM
fluorescence intensity
wavelenght, nm
Aptamer 64
RAFT concentration
0 µM
30 µM
62,5 µM
125 µM
250 µM
500 µM
750 µM
1000 µM
fluorescence intensity
wavelenght, nm
Aptamer 90
DOPC concentration
0 µM
62,5 µM
125 µM
250 µM
500 µM
750 µM
1000 µM
1500 µM
fluorescence intensity
wavelenght, nm
Aptamer 90
RAFT concentration
30 µM
62,5 µM
125 µM
250 µM
500 µM
750 µM
1000 µM
fluorescence intensity
wavelenght, nm

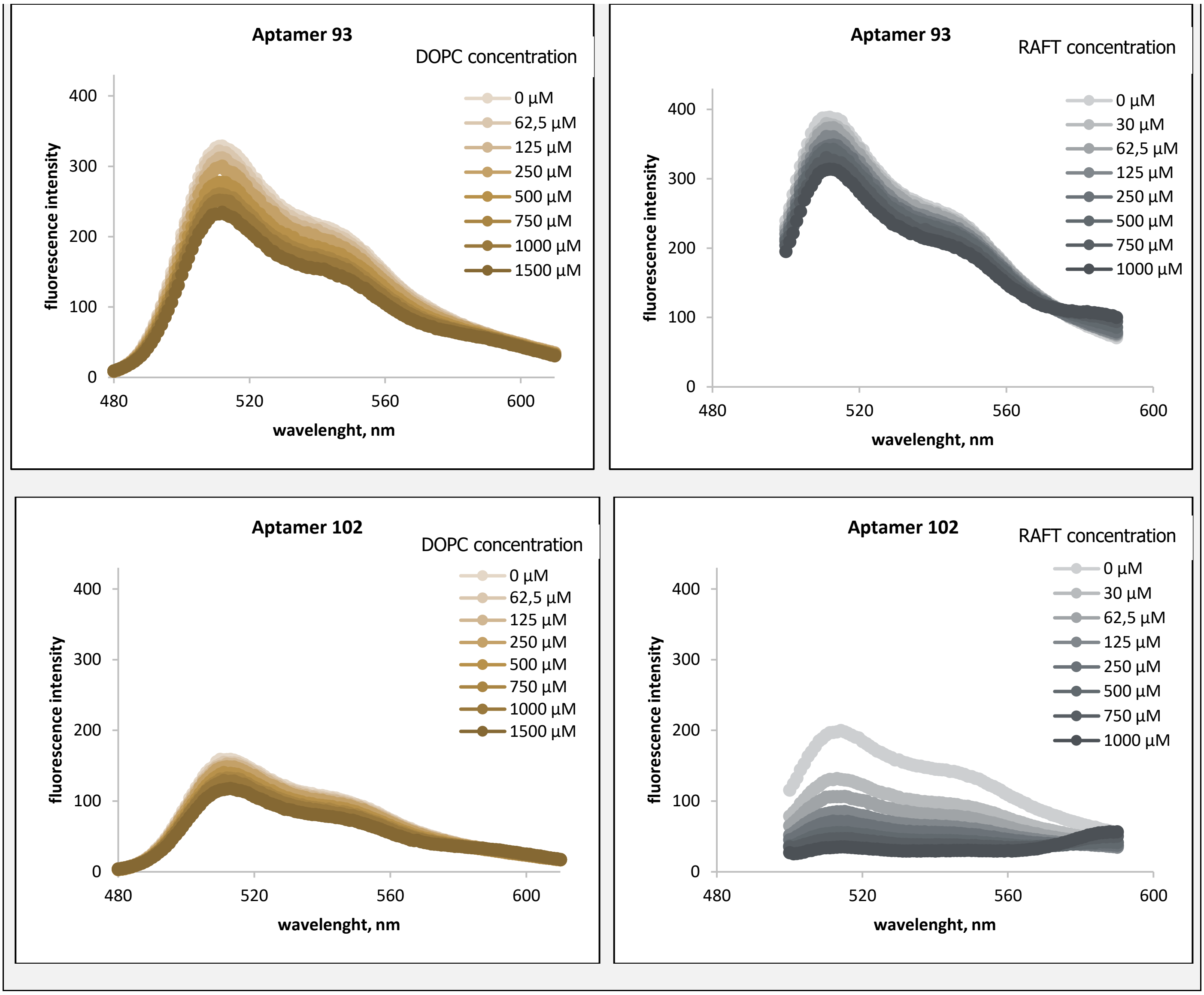
Aptamer 93
DOPC concentration
fluorescence intensity
400
300
200
100
0
480
520
560
600
wavelenght, nm
0 µM
62,5 µM
125 µM
250 µM
500 µM
750 µM
1000 µM
1500 µM
Aptamer 93
RAFT concentration
0 µM
30 µM
62,5 µM
125 µM
250 µM
500 µM
750 µM
1000 µM
Aptamer 102
DOPC concentration
Aptamer 102
RAFT concentration

**B.** Change of YOYO-1 fluorescence during titration of RNA aptamers with DOPC liposomes (left) or RAFT liposomes (right).

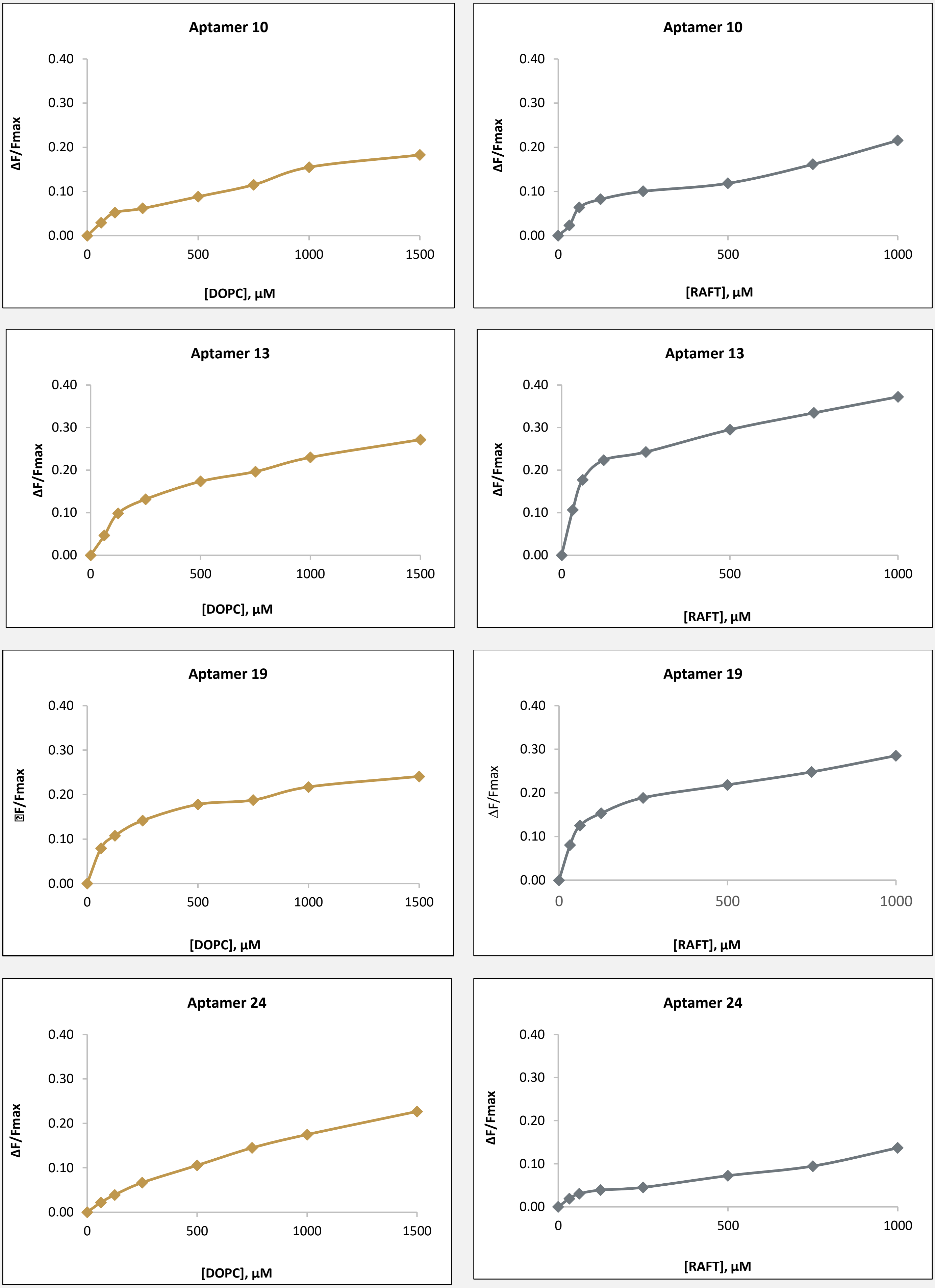

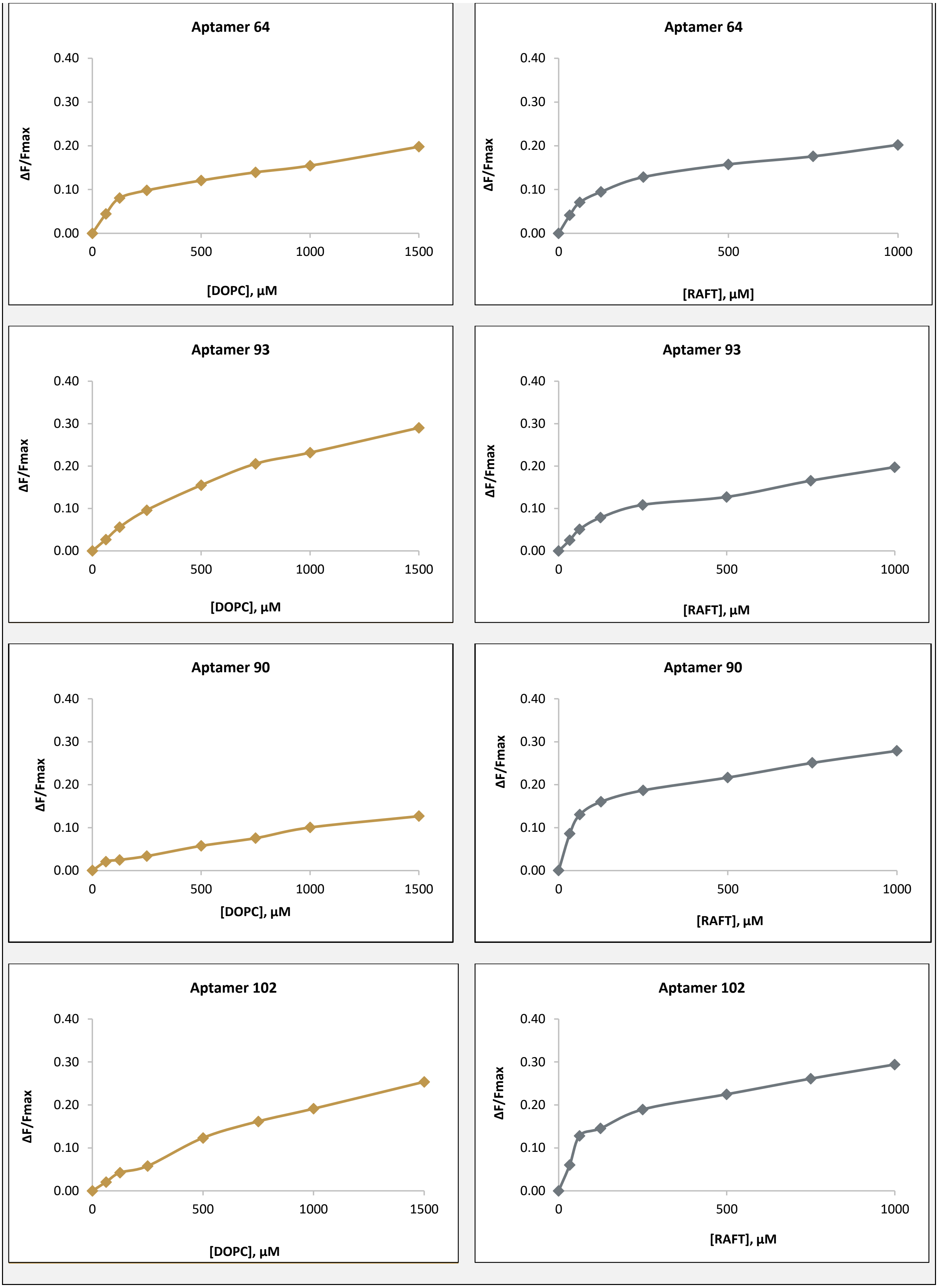

Aptamer 64
ΔF/Fmax
0.40
0.30
0.20
0.10
0.00
0
500
1000
1500
[DOPC], µM
Aptamer 64
ΔF/Fmax
0.40
0.30
0.20
0.10
0.00
0
500
1000
[RAFT], µM]
Aptamer 93
ΔF/Fmax
0.40
0.30
0.20
0.10
0.00
0
500
1000
1500
[DOPC], µM
Aptamer 93
ΔF/Fmax
0.40
0.30
0.20
0.10
0.00
0
500
1000
[RAFT], µM
Aptamer 90
ΔF/Fmax
0.40
0.30
0.20
0.10
0.00
0
500
1000
1500
[DOPC], µM
Aptamer 90
ΔF/Fmax
0.40
0.30
0.20
0.10
0.00
0
500
1000
[RAFT], µM
Aptamer 102
ΔF/Fmax
0.40
0.30
0.20
0.10
0.00
0
500
1000
1500
[DOPC], µM
Aptamer 102
ΔF/Fmax
0.40
0.30
0.20
0.10
0.00
0
500
1000
[RAFT], µM

**C.** Reciprocal of YOYO-1 fluorescence change during titration with DOPC (in left) or RAFT (right) liposomes.

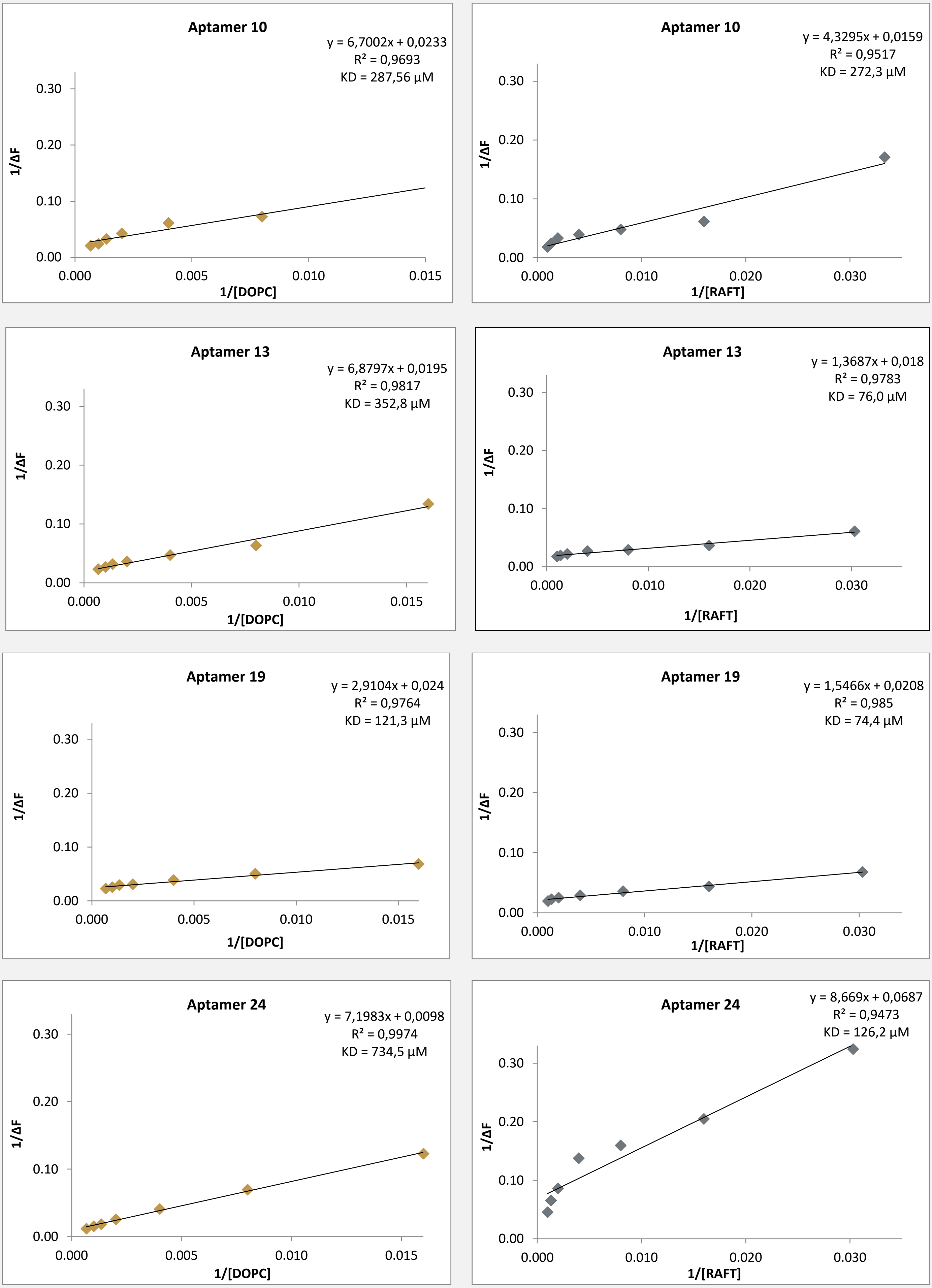

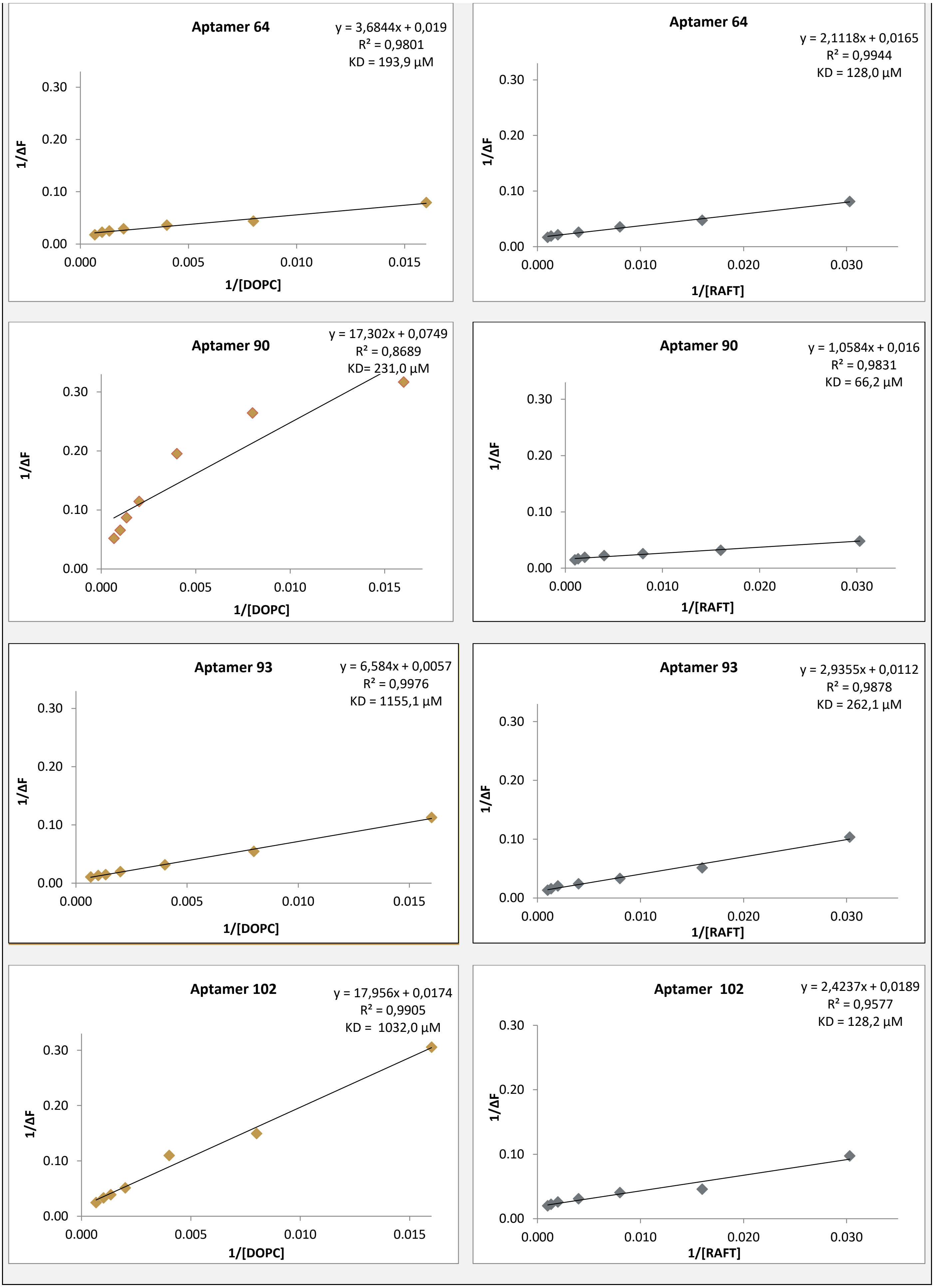

Aptamer 64
y = 3,6844x + 0,019
R² = 0,9801
KD = 193,9 µM
1/ΔF
0.30
0.20
0.10
0.00
0.000
0.005
0.010
0.015
1/[DOPC]
Aptamer 64
y = 2,1118x + 0,0165
R² = 0,9944
KD = 128,0 µM
1/ΔF
0.30
0.20
0.10
0.00
0.000
0.010
0.020
0.030
1/[RAFT]
Aptamer 90
y = 17,302x + 0,0749
R² = 0,8689
KD= 231,0 µM
1/ΔF
0.30
0.20
0.10
0.00
0.000
0.005
0.010
0.015
1/[DOPC]
Aptamer 90
y = 1,0584x + 0,016
R² = 0,9831
KD = 66,2 µM
1/ΔF
0.30
0.20
0.10
0.00
0.000
0.010
0.020
0.030
1/[RAFT]
Aptamer 93
y = 6,584x + 0,0057
R² = 0,9976
KD = 1155,1 µM
1/ΔF
0.30
0.20
0.10
0.00
0.000
0.005
0.010
0.015
1/[DOPC]
Aptamer 93
y = 2,9355x + 0,0112
R² = 0,9878
KD = 262,1 µM
1/ΔF
0.30
0.20
0.10
0.00
0.000
0.010
0.020
0.030
1/[RAFT]
Aptamer 102
y = 17,956x + 0,0174
R² = 0,9905
KD = 1032,0 µM
1/ΔF
0.30
0.20
0.10
0.00
0.000
0.005
0.010
0.015
1/[DOPC]
Aptamer 102
y = 2,4237x + 0,0189
R² = 0,9577
KD = 128,2 µM
1/ΔF
0.30
0.20
0.10
0.00
0.000
0.010
0.020
0.030
1/[RAFT]
