# Supplementary material for: Role of RNA Motifs in RNA Interaction with Membrane Lipid Rafts: Implications for Therapeutic Applications of Exosomal RNAs
Source: Int J Mol Sci. 2021 Aug 30;22(17):9416. doi: 10.3390/ijms22179416 (PMC8431113; doi:10.3390/ijms22179416)

Figure S2. The interactions of the CTB555, FAST DiI-labelled exosomes with RNA aptamers

A. Change of CTB555 and FAST DiI fluorescence during titration of exosomes with RNA aptamers

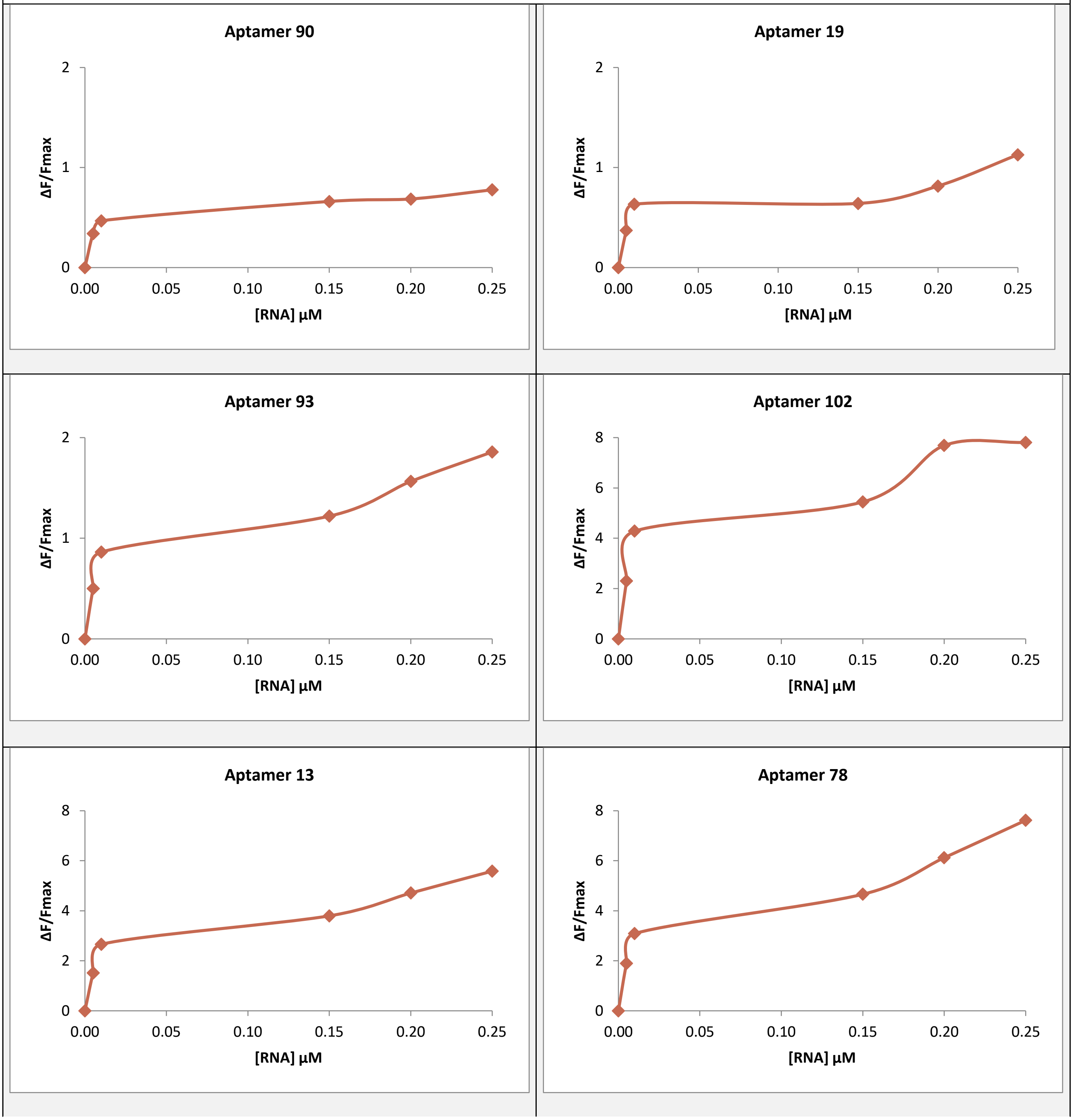

B. Reciprocal of CTB555and FAST DiI fluorescence change during titration with RNA aptamers

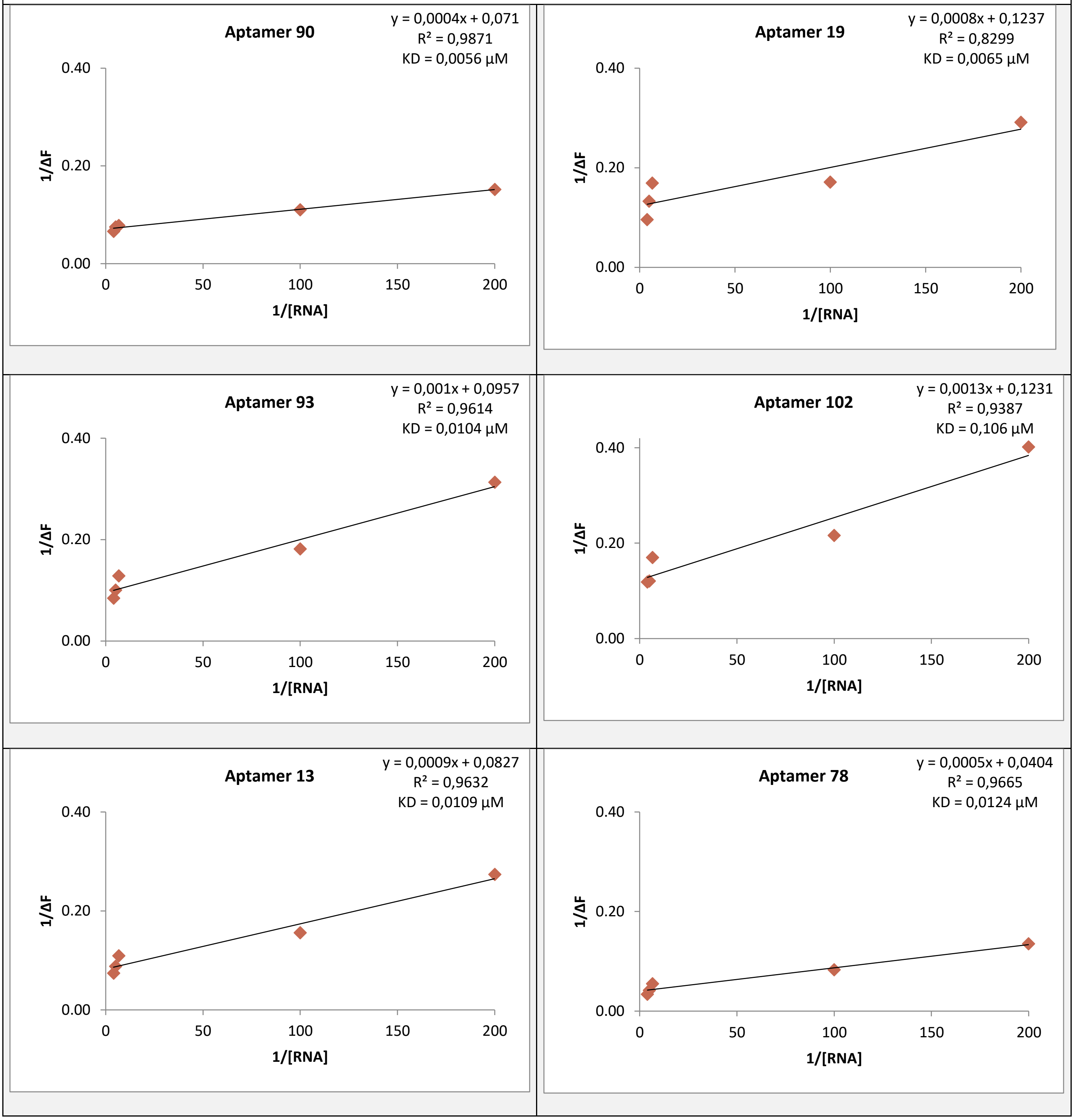

Supplement: Supplementary file 1 [file ijms-22-09416-s001.zip › ijms-1297205-Supplementary material/Figure S2.pdf]
