# Supplementary material for: Role of RNA Motifs in RNA Interaction with Membrane Lipid Rafts: Implications for Therapeutic Applications of Exosomal RNAs
Source: Int J Mol Sci. 2021 Aug 30;22(17):9416. doi: 10.3390/ijms22179416 (PMC8431113; doi:10.3390/ijms22179416)
Supplement: Supplementary file 1 [file ijms-22-09416-s001.zip › ijms-1297205-Supplementary material/Table S1.pdf]

Table S1. Location of RNA motifs in the alternative 2º structures of aptamers. Both EXO- and RAFT-motif CCCU is marked in green, other EXO-motifs (GGAG, UGAG and UCCU) are marked in red and other RAFT-motifs (UCCC, CUCC, UUGU) are marked in blue. The free energy of each structural variant is given below the aptamer structure.

RNA NAME	LOCATION OF RNA MOTIF IN THE APTAMER WITH THE LOWEST FREE ENERGY	LOCATION OF RNA MOTIFS IN THE OTHER ALTERNATIVE STRUCTURE(S) OF APTAMER 2º STRUCTURE	
**54**	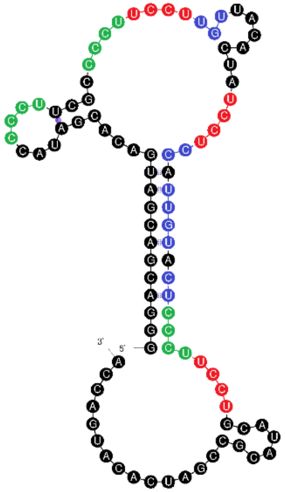 Structure 1 : ΔG = -11.20 kcal/mol	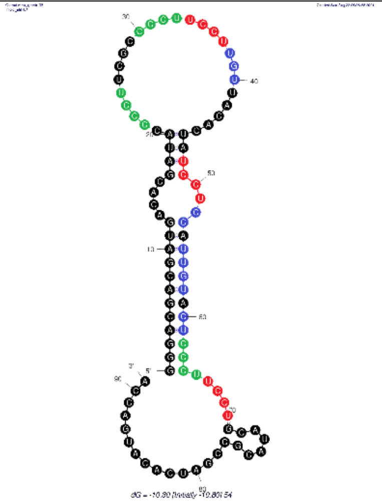 Structure 2 : ΔG = -10.80 kcal/mol,	
**90**	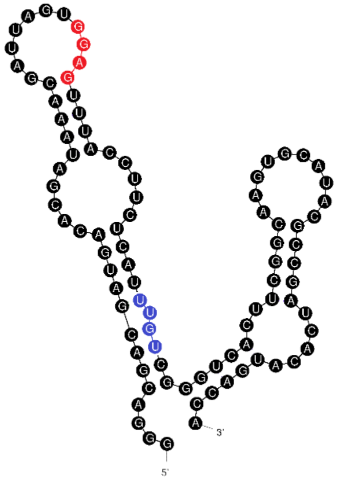 Structure 1 : ΔG = -22.90 kcal/mol	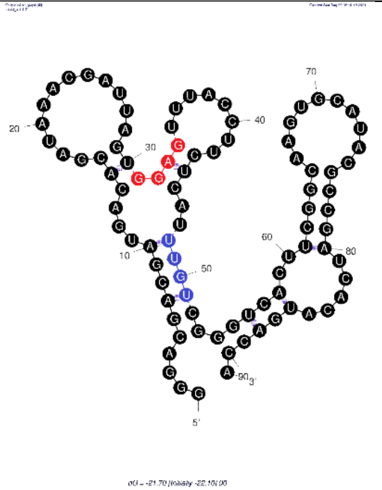 Structure 2 : ΔG = -22.10 kcal/mol	

**19**	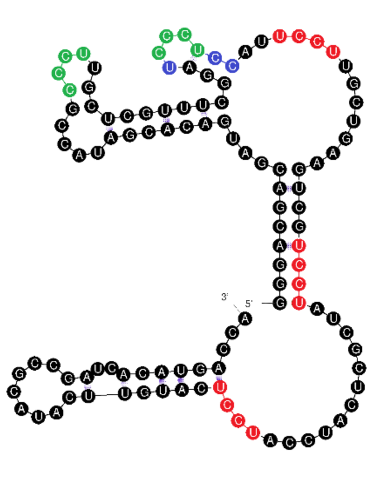<br>Structure 1 : ΔG = -23.00 kcal/mol,	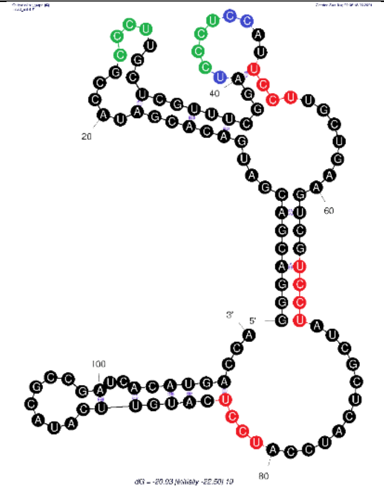<br>Structure 2 : ΔG = -22.50 kcal/mol,	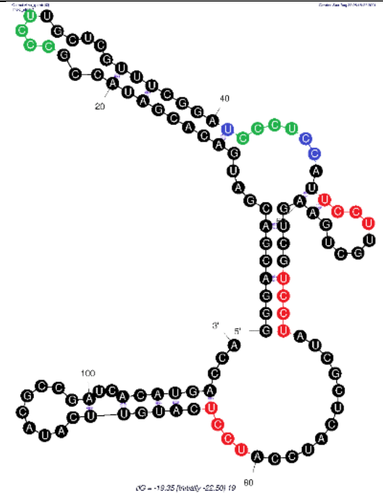<br>Structure 3 : ΔG = -22.50 kcal/mol	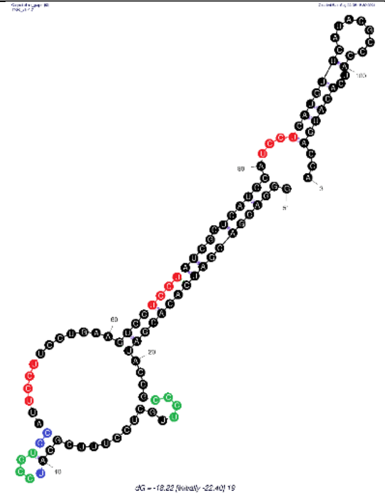<br>Structure 4 : ΔG = -22.40 kcal/mol,
**102**	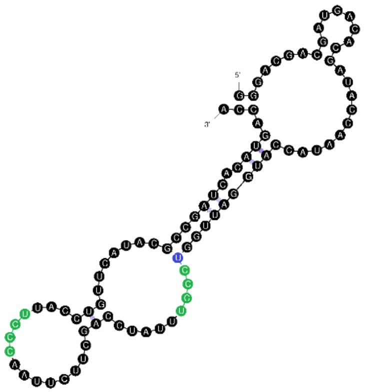<br>Structure 1 : ΔG = -12.90 kcal/mol,	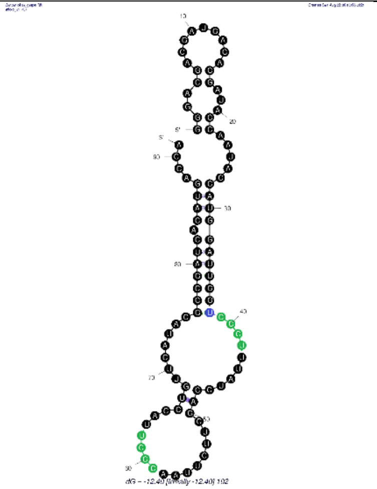<br>Structure 2 : ΔG = -12.40 kcal/mol,		

**13**	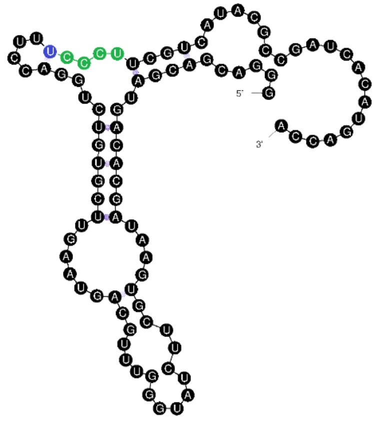<br>Structure 1 : ΔG = -20.10 kcal/mol,	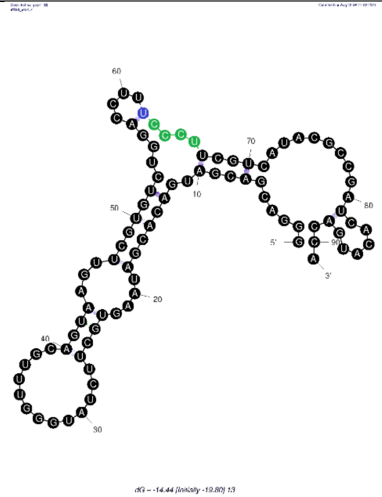<br>Structure 2 : ΔG = -19.80 kcal/mol,	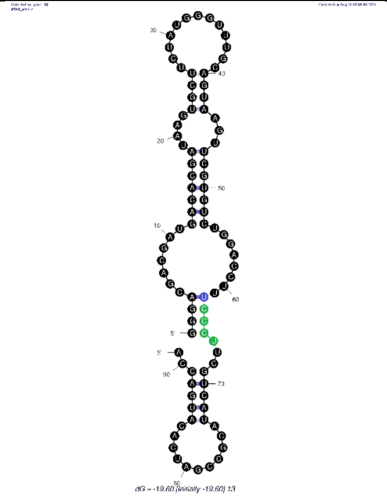<br>Structure 3 : ΔG = -19.60 kcal/mol,	
**64**	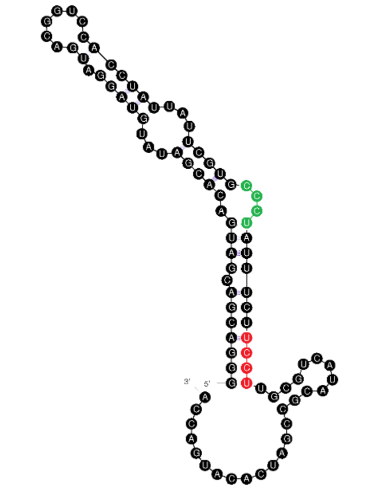<br>Structure 1 : ΔG = -20.20 kcal/mol,	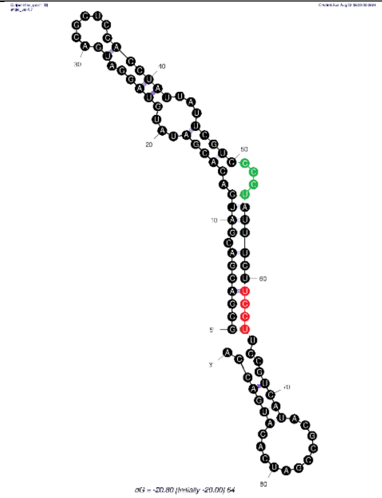<br>Structure 2 : ΔG = -20.00 kcal/mol,		

**24**	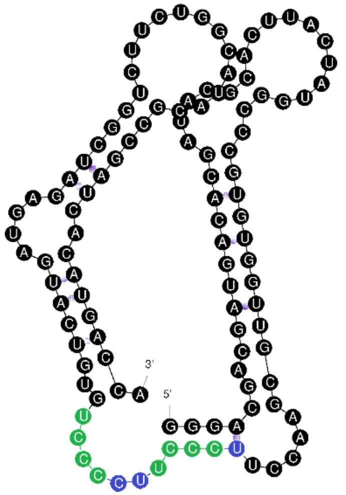 Structure 1 : ΔG = -31.40 kcal/mol,	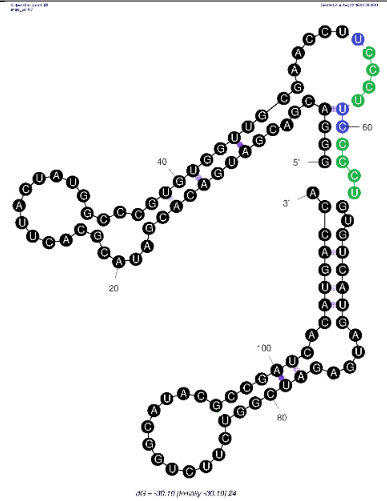 Structure 2 : ΔG = -30.10 kcal/mol,			
**93**	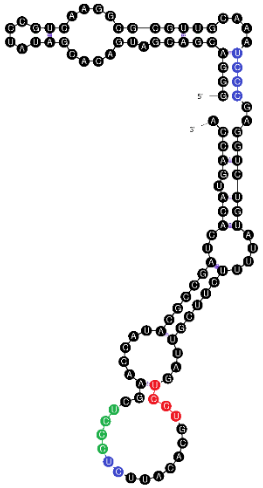 Structure 1 : ΔG = -19.20 kcal/mol,	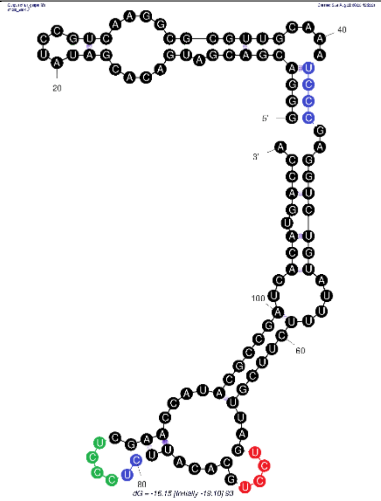 Structure 2 : ΔG = -19.10 kcal/mol,	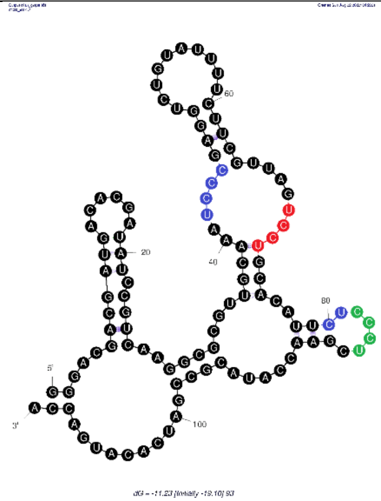 Structure 3 : ΔG = -19.10 kcal/mol,	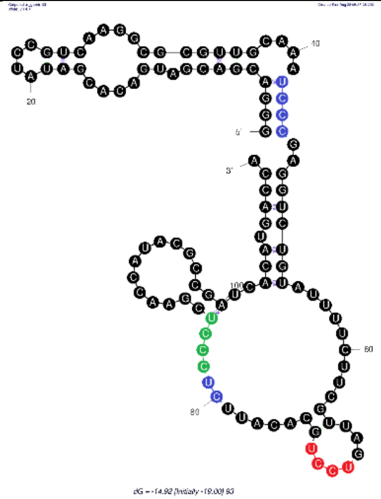 Structure 4 : ΔG = -19.00 kcal/mol,	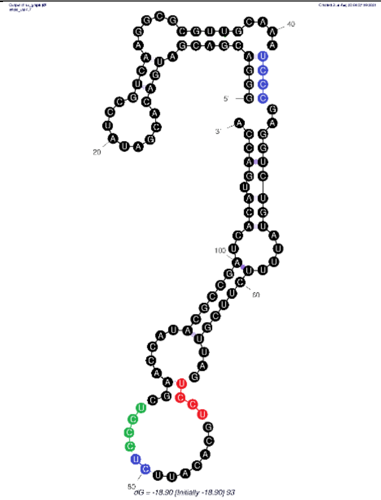 Structure 5 : ΔG = -18.90 kcal/mol,

**10**	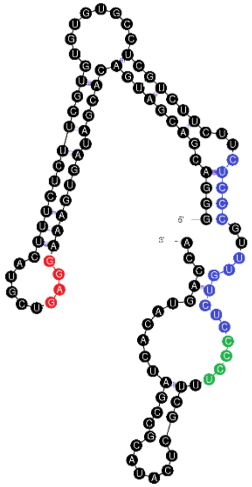 Structure 1 : ΔG = -25.90 kcal/mol,				
**78**	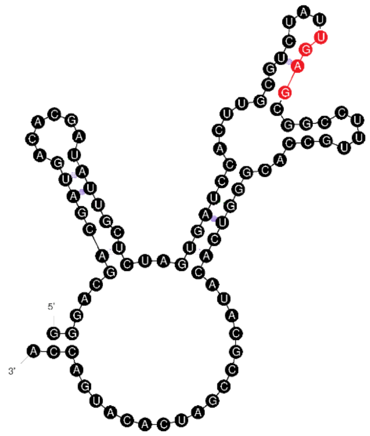 Structure 1 : ΔG = -20.00 kcal/mol,	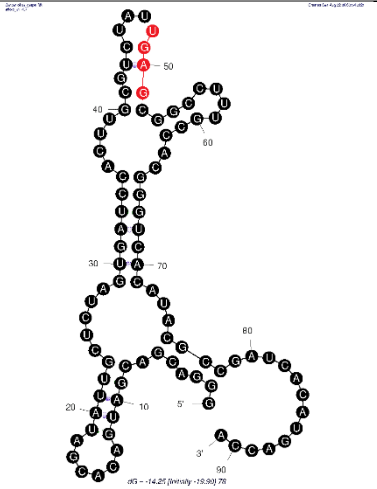 Structure 2 : ΔG = -19.90 kcal/mol,	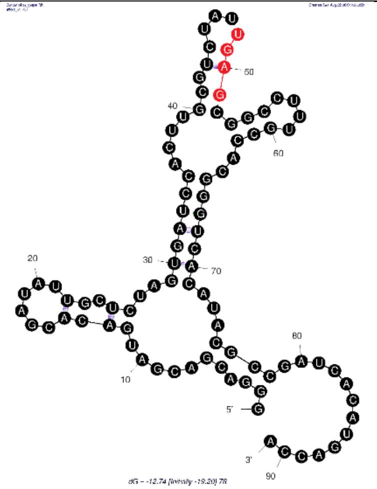 Structure 3 : ΔG = -19.20 kcal/mol,	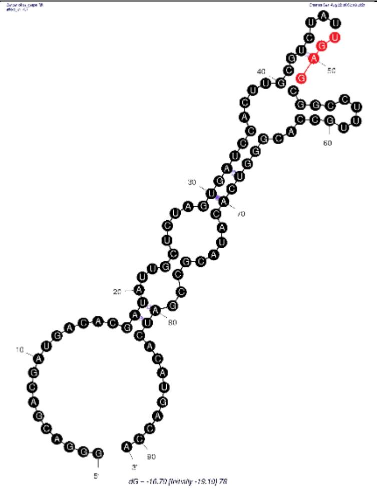 Structure 4 : ΔG = -19.10 kcal/mol,	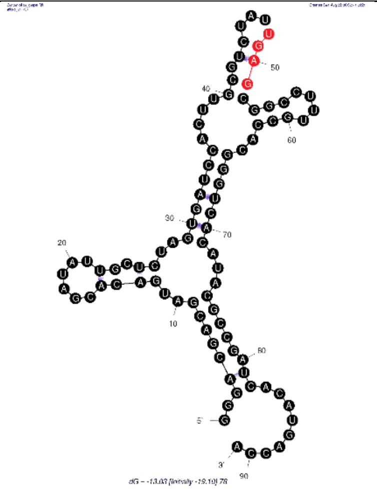 Structure 5 : ΔG = -19.10 kcal/mol,
